# Synthesis of Arginase Inhibitors: An Overview

**DOI:** 10.3390/pharmaceutics17010117

**Published:** 2025-01-16

**Authors:** Maria Cristina Molaro, Chiara Battisegola, Marica Erminia Schiano, Mariacristina Failla, Maria Grazia Rimoli, Loretta Lazzarato, Konstantin Chegaev, Federica Sodano

**Affiliations:** 1 Department of Pharmacy, “Federico II” University of Naples, 80131 Naples, Italy; mariacristina.molaro@unina.it (M.C.M.); chiara.battisegola@unina.it (C.B.); maricaerminia.schiano@unina.it (M.E.S.); rimoli@unina.it (M.G.R.); 2 Department of Drug Science and Technology, University of Turin, 10125 Turin, Italy; mariacristina.failla@unito.it (M.F.); loretta.lazzarato@unito.it (L.L.); konstantin.chegaev@unito.it (K.C.)

**Keywords:** Arginase, Arginase inhibitors, synthetic protocols

## Abstract

Arginase (ARG) is a binuclear manganese-containing metalloenzyme that can convert L-arginine to L-ornithine and urea and plays a key role in the urea cycle. It also mediates different cellular functions and processes such as proliferation, senescence, apoptosis, autophagy, and inflammatory responses in various cell types. In mammals, there are two isoenzymes, ARG-1 and ARG-2; they are functionally similar, but their coding genes, tissue distribution, subcellular localization, and molecular regulation are distinct. In recent decades, the abnormal expression of ARG-1 or ARG-2 has been reported to be increasingly linked to a variety of diseases, including cardiovascular disease, inflammatory bowel disease, Alzheimer’s disease, and cancer. Therefore, considering the current relevance of this topic and the need to address the growing demand for new and more potent ARG inhibitors in the context of various diseases, this review was conceived. We will provide an overview of all classes of ARG inhibitors developed so far including compounds of synthetic, natural, and semisynthetic origin. For the first time, the synthesis protocol and optimized reaction conditions of each molecule, including those reported in patent applications, will be described. For each molecule, its inhibitory activity in terms of IC_50_ towards ARG-1 and ARG-2 will be reported specifying the type of assay conducted.

## 1. Introduction

Arginase (ARG) has roots in early life forms. It is a manganese-containing binuclear metalloenzyme capable of converting L-arginine to urea and L-ornithine. Urea provides protection against ammonia (NH_3_), while L-ornithine serves to stimulate cell growth and other physiological functions. This interconnection with various metabolic pathways, such as polyamine synthesis and energy metabolism regulation, underscores the importance of ARG in maintaining metabolic homeostasis [1]. L-arginine is one of the most versatile amino acids in animal cells, serving as a precursor not only for the protein synthesis but also for the production of nitric oxide, urea, polyamines, proline, glutamate, creatine, and agmatine [2]. In mammals, there are two isoenzymes: arginase-1 (ARG-1) and arginase-2 (ARG-2). They are functionally similar, but the coding genes, tissue distribution, subcellular localization, and molecular regulation are distinctive. ARG-1 is localized in the cytoplasm and mainly expressed in the liver, where it is responsible for the detoxification of ammonia in the urea cycle [1]. In contrast, ARG-2 is predominantly found in the mitochondria and is primarily involved in polyamine generation [3]. Both enzymes metabolize L-arginine, which is severely depleted in the immunosuppressive tumour microenvironment (TME), where it is crucial for proliferating cells. This positions them at the forefront of immune escape through various mechanisms.

Within the TME, ARG-1 plays a crucial role in immune evasion by activating immunosuppressive cells, such as myeloid-derived suppressor cells (MDSCs), a heterogeneous population of bone marrow-derived cells that suppress immune responses and facilitate tumour progression [4]. In addition to high expression in MDSCs, ARG-1 is also upregulated in tumour-associated macrophages (TAMs) and activated neutrophils within the TME [5,6]. In these cells, it depletes L-arginine, impairing T-cell activation and proliferation, ultimately enabling cancer cells to evade immune surveillance.

Recently, ARG-2 has received special attention. It has been reported that the ARG-2 pathway is a means by which regulatory T cells (Tregs), also called CD_4_^+^ cells, regulate inflammation in tissues. Compared to psoriatic Tregs, healthy Tregs express almost 4-fold more ARG-2; similarly, Tregs in metastatic melanoma lesions express high levels of ARG-2 protein, with these levels being higher in tumour-infiltrating Tregs than in Tregs isolated from healthy skin [7]. ARG-2 reduces the survival and proliferation of effector T cells (CD_8_^+^), thereby impacting antitumour immune responses. Thus, L-arginine metabolism via ARG-2 is an immunoregulatory pathway used by Tregs in human tissues, with significant implications for both autoimmunity and cancer.

The disturbance in the expression of ARG can lead to a range of vascular, neurological, immunological, and inflammatory disorders, as the isoforms ARG-1 and ARG-2 are involved in the regulation of nitric oxide (NO), polyamines, proline, and, particularly in the immune system, endothelial cells, and neuronal cells [8,9,10].

Regarding the involvement of ARG in the immune system, in the presence of an inflammatory stimulus, the enzyme nitric oxide synthase (NOS) produces NO. This NO interacts with reactive oxygen species (ROS), creating a cytotoxic nitrosative stress environment that inhibits both cellular replication and pathogenic activity [11]. On the other hand, the enzyme ARG metabolizes L-arginine, regulating defence mechanisms and downregulating NO production. This helps prevent uncontrolled cellular apoptosis triggered by ONOO^–^ species generated from excess NO reacting with superoxide radicals (O_2_^−^) [12].

An altered balance in the expression of these two enzymes can cause serious issues for the immune system. Additionally, the dysregulated release of ARG from cells and tissues into extracellular fluids can further compromise the defence mechanisms of macrophages against pathogens by limiting the bioavailability of L-arginine, reducing NO levels, and disrupting cytokine production pathways. It has been shown that ARG is implicated in disorders such as multiple sclerosis, as the increased regulation of the enzyme ARG-2 stimulates the production of cytokines that differentiate T helper 17 cells, thus inducing inflammation [13]. In studies on obesity-induced vasculopathy, it has been observed that high levels of fats and sucrose activate Rho-associated kinases, increasing the expression of ARG-1. The increased synthesis of polyamines mediated by ARG-1 promotes cellular proliferation and fibrosis; additionally, the rise in ROS levels contributes to dysfunction [14].

The enzyme ARG, particularly its isoform ARG-2, plays a crucial role in maintaining the balance of the cardiovascular system by regulating the levels of NO [15]. This helps reduce oxidative damage to the endothelium, promotes blood vessel dilation, and prevents the adhesion and aggregation of leukocytes and platelets. An imbalance between the enzymes that degrade L-arginine (ARG and NOS) can contribute to many age-related cardiovascular complications, such as vascular stiffness, ventricular hypertrophy, hypertension, inflammation, and disorders caused by oxidative stress [16]. Studies have shown that a high-fat, high-cholesterol diet causes liver damage in mice, leading to the overexpression of ARG-1, a consequent reduction in circulating L-arginine levels, and cardioprotective effects mediated by NO [17]. In contrast, a glucose-free diet in mice results in increased expression of ARG-2, triggering signalling pathways that protect hepatocytes from fat accumulation, inflammatory responses, insulin resistance, and glucose intolerance [18].

ARG and NOS are present in both the peripheral and central nervous systems, but their interaction is complicated by the complexity of the brain. ARG is essential for the detoxification of ammonia and the synthesis of polyamines, which are necessary for neuronal development and regeneration. NO, on the other hand, is a neurotransmitter that contributes to synaptic plasticity and cerebral blood flow. During development, high levels of cAMP stimulate ARG-1 to promote neuronal survival. However, with ageing, an imbalance between ARG and NOS can reduce NO production, contributing to neurodegenerative diseases [19].

One neurodegenerative disease associated with the alteration of ARG enzyme expression is Alzheimer’s disease. It has been observed that microglial activation leads to the production of cytokines, which induce increased expression of ARG-1 and ARG-2 in the brain. However, the accumulation of ARG-2 at sites of β-amyloid deposition causes the uncoupling of NOS, generating O_2_^−^ and neurodegenerative oxidative stress [20]. This may be an attractive molecular imaging target for the evaluation of Alzheimer’s disease progression [21]. In certain contexts, an excess of NO can cause neuronal damage and brain trauma due to excitotoxicity. Notably, the role of ARG-2 is completely reversed in neurodegenerative diseases compared to cardiovascular disorders, highlighting the importance of understanding the distinct contributions of each isoform in different disease contexts. This underscores the need for selective molecules tailored to each isoform.

In line with the significant role of ARG-1 and ARG-2 in numerous diseases, there has been growing interest in developing inhibitors for these enzymes. In particular, identifying the ARG enzymes as critical metabolic checkpoints in the TME has spurred the design of novel ARG inhibitors. Both natural and synthetic compounds have been evaluated in various in vitro, ex vivo, and in vivo models [22,23]. Natural compounds like chlorogenic acid and picetannol, as well as synthetic compounds such as L-arginine-like derivatives and boronic acid derivatives, have been studied. These boronic acids are α-amino acids with a lateral boronic group capable of chelating the two manganese ions required for enzymatic activity. However, their inhibitory activity (IC_50_) was in the micromolar range, indicating the need for further improvement. The initial approach taken by Van Zandt et al., later upgraded by others, focused on expanding 2-(S)-amino-6-boronohexanoic acid (ABH) with moieties capable of additional interactions with residues Asp181, Asp183, and Asp202 [24,25,26]. Further improvements in the pharmacological profile were achieved by limiting the conformational flexibility of ABH. Notable examples include CB-1158 (numidargistat) and OATD-02 [27,28]. In a recent study, OATD-02 demonstrated superior in vivo antitumor capacity compared to CB-1158, as it inhibited both extracellular ARG-1 within the TME and cellular ARG-2 overexpressed in the TME [29]. By inhibiting ARG-2, OATD-02 was found to regulate the activity of CD_8_^+^ cells and Tregs, thus controlling a key player responsible for the metabolic adaptation typical of hypoxic tumours.

**Figure 1:**
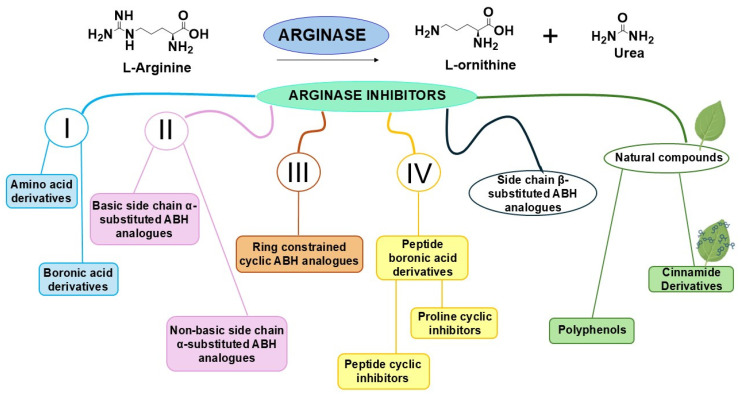
Schematic representation of all ARG inhibitor classes.

This review aims to provide a comprehensive overview of ARG inhibitors, focusing on the ongoing search for these molecules involved in multiple key pathophysiological processes. We categorize these inhibitors into generations and classes, as illustrated in Figure 1. For each molecule, its inhibitory activity (IC_50_) against ARG-1 and ARG-2 will be reported, with colorimetric assays used for all compounds unless otherwise specified.

Three reviews have previously addressed this topic, each from a different perspective. Borek et al. focused on the SAR and pharmacokinetic properties of each inhibitor [22]; Niu et al. discussed the underlying mechanism of ARG in tumour cell growth and summarized recent clinical research on ARG targeting for cancer therapy [30]. Clemente et al. explored the potential of ARG as a molecular imaging biomarker, stimulating the development of high-affinity, specific ARG imaging probes [16]. Failla et al. analysed the structural characteristics and plasticity of ARG-1 and ARG-2 binding sites, aiming to design inhibitors with new binding patterns [31]. However, none of these reviews have addressed the synthetic procedures of ARG inhibitors, a critical aspect from a medicinal chemistry perspective.

The chemical synthesis of these inhibitors presents numerous challenges, primarily due to their inherent chemical complexity. For instance, replacing the guanidine group with a boronic acid residue has proven effective. This modification preserves the geometry and electrophilicity of the original group while improving the compound’s physicochemical properties, such as reduced polarity and non-basicity. However, synthesizing a boronic acid derivative is challenging due to the reactivity of the functional group, necessitating the use of a protected ester form. Although using a protecting group can simplify the synthetic process, this is not always the case with amino acid derivatives, such as ARG inhibitors. These derivatives must mimic the amino acid L-arginine, requiring both amino and carboxylic groups. Given that these functional groups are as reactive as boronic acid, selecting the optimal protecting group and synthetic strategy is challenging. The inherent chemical complexity of these procedures presents scalability issues and challenges with reproducibility, further complicated by the presence of chiral centres and the need for stereoselective syntheses.

In this review, we provide a complete overview of all classes of ARG inhibitors, including molecules of synthetic and natural origins. For the first time, we also present the synthetic protocols and the optimized reaction conditions for each molecule. Many structures and syntheses of ARG inhibitors have been reported in patents that are often difficult to access and interpret. This review aims to be a comprehensive guide to the synthesis of ARG inhibitors, addressing critical issues from a synthetic chemical perspective and suggesting how these can be overcome through medicinal chemistry strategies.

## 2. First-Generation Inhibitors

The first ARG inhibitors were developed by analysing the chemical structure of the enzyme’s natural substrates, such as L-arginine and other structurally similar amino acids. The enzyme’s small and highly polar active site favours the accommodation of amino acids with a natural L-configuration (such as L-arginine analogues), which is also the configuration responsible for the enzyme’s activity.

The subsequent inclusion of a boronic group into the inhibitor structures was crucial for two main reasons. First, during the binding mechanism with the ARG enzyme, the boronic group forms a tetrahedral boronate anion by incorporating an OH group, effectively mimicking the transition state of the hydrolysis of the trigonal planar guanidine group in L-arginine by ARG. Second, the boron atom’s electron deficiency facilitates nucleophilic attack by the hydroxide ion, enhancing the activity of the inhibitor. Boronic acid also serves as an excellent guanidine substitute due to its similar geometry and electrophilicity, while offering better physicochemical properties—it is less polar, non-basic, and has fewer hydrogen bond donors [32]. In addition to incorporating the boronic moiety, other structural modifications included varying the length of the alkyl side chain, introducing a sulphur atom along the carbon chain, and adding a phenyl ring to the side chain to create a conformationally restricted analogues. These modifications led to the development of ARG inhibitors, which, divided into α-amino acid derivatives and boronic acid-containing compounds, constitute the class of first-generation inhibitors [22].

### 2.1. α-Amino Acid Derivatives

**Figure 2:**
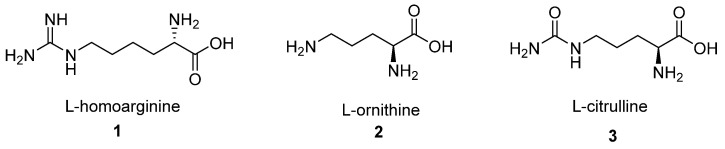
Chemical structures of compounds **1**–**3**.

ARG inhibitors derived from amino acids include L-homoarginine (**1**), L-ornithine (**2**), and L-citrulline (**3**) categorized as “natural amino acids compounds” (Figure 2). L-homoarginine interacts slowly with ARG, as the enzyme’s hydrolytic efficiency depends on the side chain of the α-amino acid substrate. It inhibits human hARG-1 with IC_50_ and K_i_ values of 8.14 ± 0.52 mM and 6.1 ± 0.50 mM, respectively, and hARG-2 with IC_50_ and K_i_ values of 2.52 ± 0.01 mM and 1.73 ± 0.10 mM, respectively. ARG activity was assessed by measuring L-ornithine formation in HEK293T cell lysates [33]. L-ornithine shows a significant inhibition of rat ARG, achieving 85.9% inhibition at 10 mM. Similarly, L-citrulline exhibits inhibition levels comparable to L-ornithine, with a bovine liver ARG inhibition of 60% at 20 mM for L-ornithine and 53% at 20 mM for L-Citrulline, as measured by a [^14^C] urea assay [34,35]. The IC_50_ values, in the millimolar range, obtained with the aforementioned α-amino acid derivatives in in vitro models indicated that they were weak ARG inhibitors, predicting an even weaker therapeutic potential in vivo.

**Scheme 1:**
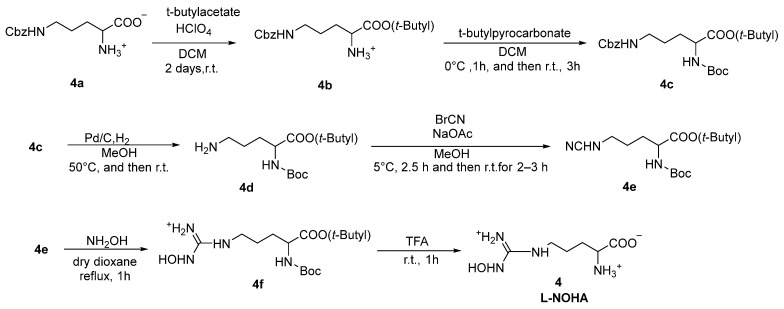
Synthesis of L-NOHA (**4**).

N^ω^-hydroxyarginine (**L-NOHA**, **4**), is a hydroxylated derivative of L-arginine and a potent ARG inhibitor. It demonstrated the following activity against hARG with the following results: hARG-1 with a *K*_d_ of 3.6 μM (pH 8.5, surface plasmon resonance, SPR) and hARG-2 with *K*_i_ values of 1.6 μM, (pH 7.5) and 2 μM (pH 9.5, radioactive assay) [36,37]. For the synthesis of L-NOHA, Wallace et al. adopted Bodanszky’s method (Scheme 1) [38,39]. Starting with *N*^δ^-(benzyloxycarbonyl)-L-ornithine (**4a**), the carboxylic acid group was protected using *tert*-butylacetate and HClO_4_, yielding the corresponding *tert*-butyl ester (**4b**). Compound **4b** was treated with *tert*-butyl pyrocarbonate in dichloromethane (DCM) at 0 °C and then at room temperature (r.t.) to protect the amine group, producing compound **4c.** Next, hydrogenation with H_2_ and Pd/C removed the benzyloxycarbonyl group, generating the *N^α^*-(*tert*-butyroxycarbonyl)-L-ornithine tert-butyl ester (**4d**) [39]. To form the cyanamide derivative **4e**, the reaction of **4d** with BrCN in MeOH using NaOAc as a base (Bailey’s method) was performed [40]. The subsequent reaction of **4e** with NH_2_OH in dry dioxane under reflux produced the *N^α^*-(*tert*-butyloxycarbonyl)-*N^ω^*-hydroxy-L-arginine *tert*-butyl ester (**4f**). Finally, deprotection of **4f** with trifluoroacetic acid (TFA) yielded *N^ω^*-hydroxyarginine (**4**) [39].

**Scheme 2:**
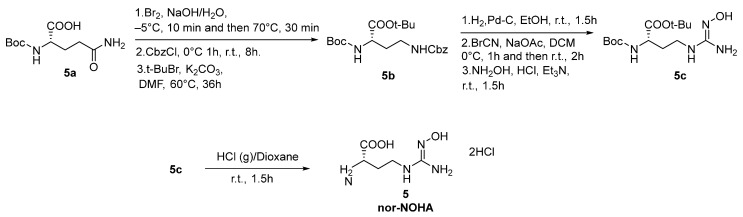
Synthesis of compound **5**.

**Nor-NOHA** (**5**), a hydroxyguanidine derivative with a shorter alkyl chain, is another compound that effectively inhibits the enzyme ARG [41]. It was tested on hARG with the following results: on hARG-1 (pH 8.5) it showed a *K*_d_ of 0.517 μM (SPR) and 0.047 μM (isothermal titration colorimetry determination, ITC), while on hARG-2 it exhibited a *K*_i_ of 51 nM (pH 7.5). As shown by the data from the ARG inhibition tests, the smaller derivative nor-NOHA demonstrated higher affinity compared to L-NOHA and also exhibited a better pharmacokinetic profile, including improved bioavailability and faster elimination. The synthesis of nor-NOHA, which is very similar to that of L-NOHA, is outlined in Scheme 2 [36,37].

The synthesis of nor-NOHA (**5**), began with *N^α^*-*tert*(butyloxycarbonyl)-L-glutamine (**5a**) as the starting material [42]. After the Hofmann degradation of the carboxamide, the amine group was protected using benzyl chloroformate (CbzCl), followed by the protection of the carboxyl group with a *tert*-butyl ester, yielding *N^α^*-*tert*(butyloxycarbonyl)-*N^γ^*-benzyloxycarbonyl-L-2,4-*tert*-butyl-diaminobutyrate (**5b**). This intermediate **5b** underwent further reactions, including catalytic hydrogenation to selectively deprotect the amine group, followed by treatment with BrCN and NH_2_OH-HCl to introduce the hydroxyguanidine group, forming *N^α^*-*tert*(butyloxycarbonyl)-*N^ω^*-hydroxy-nor-L-*tert*-butyl arginine (**5c**). These steps were streamlined into two stages. Finally, dry HCl removed all protecting groups, yielding the target compound, nor-NOHA^.^2HCl (**5**) [43].

Two additional first-generation inhibitors, *N^ω^*-hydroxy-indospicin (**6**) and 4-hydroxy-amino-D,L-phenylalanine (**7**), are analogues of L-NOHA in which the hydroxyguanidine group is replaced by hydroxyamidine. Both have shown micromolar activity against bovine liver ARG ([^14^C] urea assay) with IC_50_ values of 50 ± 10 μM for compound **6** and 230 ± 5 μM for compound **7** [41].

**Scheme 3:**
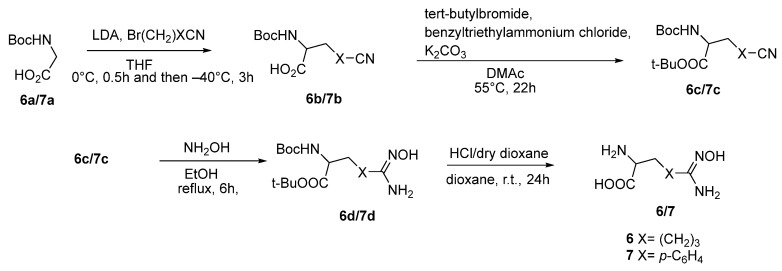
Synthesis of compounds **6** and **7**.

The synthesis of compounds **6** and **7** (see Scheme 3) has been described by Vadon et al. Briefly, *N*-BOC-glycine was alkylated using 5-bromopentanenitrile (for compound **6**) or 4-(bromomethyl)benzonitrile (for compound **7**) in dry tetrahydrofuran (THF) using lithium diisopropylamide (LDA). The resulting products, *N^α^*-*tert*-butoxycarbonyl-6-cyano-D,L-norleucine (**6b**) and *N^α^*-*tert*-butoxycarbonyl-p-cyano-D,L-phenylalanine (**7b**) had their carboxyl groups protected as a *tert*-butyl ester using benzyltriethylammonium chloride, K_2_CO_3_, and *tert*-butyl bromide in dimethylacetamide (DMAc). The intermediates *N^α^*-*tert*-butoxycarbonyl-6-cyano-D,L-norleucine-*tert*-butylester (**6c**) and *N^α^*-*tert*-butoxycarbonyl-*p*-cyano-D,L-phenylalanin-*tert*-butylester (**7c**) were refluxed with ethanolic hydroxylamine for 6 h to form the hydroxiimidine derivatives *N^α^*-*tert*-butoxycarbonyl-*N^ω^*-hydroxy-D,L-indospicine-*tert*-butylester (**6d**) and *N^α^*-*tert*-butoxycarbonyl-*p*-hydroxyamidino-D,L-phenylalanine-*tert*-butylester (**7d**). In the final synthetic step, treatment with HCl in dry dioxane at r.t. for 24 h removed the protecting groups, producing the final compounds *N^ω^*-hydroxy-D,L-indospicine (**6**) and 4-hydroxy-amino-D,L-phenylalanine (**7**), respectively [44].

**Scheme 4:**
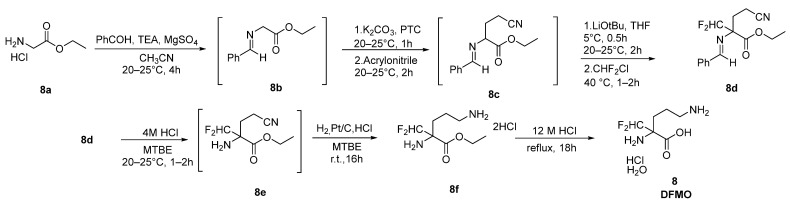
Synthesis of compound **8**.

α-Difluoromethylornithine (**DFMO**, **8**) is an irreversible inhibitor of ornithine decarboxylase (ODC), the enzyme involved in polyamines biosynthesis [34,45]. It also exhibited weak inhibitory activity against intestinal ARG, with a *K*_i_ of 3.9 ± 1.0 mM in intact HT-29 cells and 80 ± 3% inhibition at 10 mM on bovine liver arginase [46]. The synthesis of DFMO is shown in Scheme 4 [47]. The process began with glycine ethyl ester hydrochloride salt (**8a**) reacting with benzaldehyde in the presence of magnesium sulphate, acetonitrile (ACN), and triethylamine (TEA) to form the 2-benzylideneamino glycine ethyl ester (**8b**). This intermediate was then treated with acrylonitrile in the presence of K_2_CO_3_ and triethylbenzylammonium chloride, yielding ethyl 2-benzylideneamino-4-cyanobutyrate (**8c**). Next, **8c** reacted with chlorodifluoromethane and lithium *tert*-butoxide in THF at 40 °C producing ethyl-2-benzylideneamino-2-difluoromethyl-4-cyanobutyrate (**8d**). Compound **8d** was then deprotected with 4 M HCl in methyl-*tert*-butyl ether (MTBE) to give ethyl-2-amino-2-difluoromethyl-4-cyanobutyrate (**8e**), which underwent catalytic hydrogenation in MTBE and 12 M HCl to form ethyl 2,5-diamino-2-difluoromethylpentanoate dihydrochloride (**8f**). In the final step, **8f** was deprotected with 12 M HCl, yielding DFMO monohydrochloride monohydrate (**8**).

### 2.2. Boronic Acid Derivatives

**Scheme 5:**
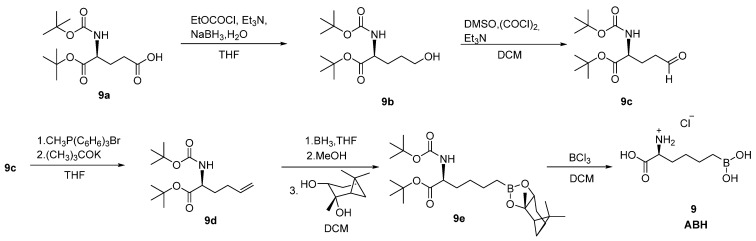
Synthesis of compound **9**.

Another class of first-generation ARG inhibitors consists of boronic acid derivatives. The compound 2(S)-amino-6-boronohexanoic acid (**ABH**, **9**) is the first boronic acid-based arginine isostere [48]. ABH exhibited strong activity, with a K_d_ of 5 nM for hARG-1, a K_i_ of 8.5 nM for hARG-2, and an IC_50_ of 0.8 μM for rat liver ARG-1 [22]. The synthesis of **ABH** is outlined in Scheme 5 [48,49]. First, (R)-5-(tert-butoxy)-4-((tert-butoxycarbonyl)amino)-5-oxopentanoic acid (**9a**) was reduced using sodium borohydride and ethyl chloroformate to produce a primary alcohol derivative (**9b**). In the next step, **9b** underwent Swern oxidation to form an aldehyde **9c**, which was used directly in a Wittig reaction with triphenylphosphonium methylilide, yielding olefin (**9d**). The hydroboration of **9d**, followed by treatment with MeOH and protection with (1S,2S,3R,5S)-(+)-pinanediol, yields intermediate **9e**. The final step involved complete deprotection using BCl_3_, yielding **ABH** (**9**) as a white semi-crystalline solid.

**Scheme 6:**
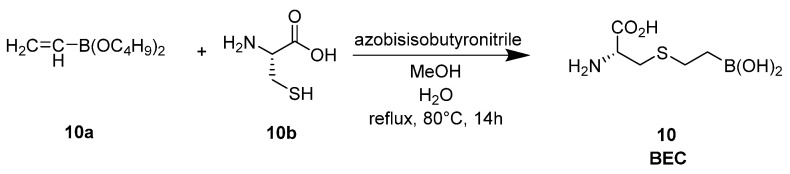
Synthesis of compound **10**.

2-Boronoethyl-L-cysteine (**BEC**, **10**) is an analogue of ABH, where a sulphur atom replaces carbon in the main chain. BEC exhibited activity with a K_d_ of 270 nM for hARG-1 (ITC) and a K_i_ of 30 nM for hARG-2 (radioactive assay) [22]. The synthesis of **BEC** (Scheme 6) involved a single-step reaction [50]. A solution of cysteine (**10b**) and dibutylenboronate (**10a**) in MeOH and H_2_O was refluxed at 80 °C under N_2_ for 14 h. Afterward, azobisisobutyronitrile was added, yielding the desired compound **10**.

ABH and BEC exhibited higher affinity for ARG than α-amino acid derivatives. However, their bioavailability was relatively low, with undetectable plasma levels, likely due to their limited ability to cross biological membranes. Additionally, their unacceptable toxicity to normal cells, stemming from their chemical reactivity and instability, rendered them unsuitable for clinical use in cancer treatment. As a result, a structural optimization study, along with the exploration of medicinal chemistry strategies—such as drug delivery systems—became crucial. Approaches aimed at enhancing the permeability and stability of these ARG inhibitors, potentially through carrier or bioprecursor prodrug strategies, are urgently needed.

## 3. Second-Generation Inhibitors

Second-generation inhibitors include α-substituted ABH analogues, which have shown promising and potent ARG inhibitors. These inhibitors are further divided into two subclasses: basic side chain α-substituted ABH analogues (see Section 3.1) and non-basic side chain α-substituted ABH analogues (see Section 3.2) [16].

### 3.1. Basic Side Chain α-Substituted ABH Analogues

**Scheme 7:**
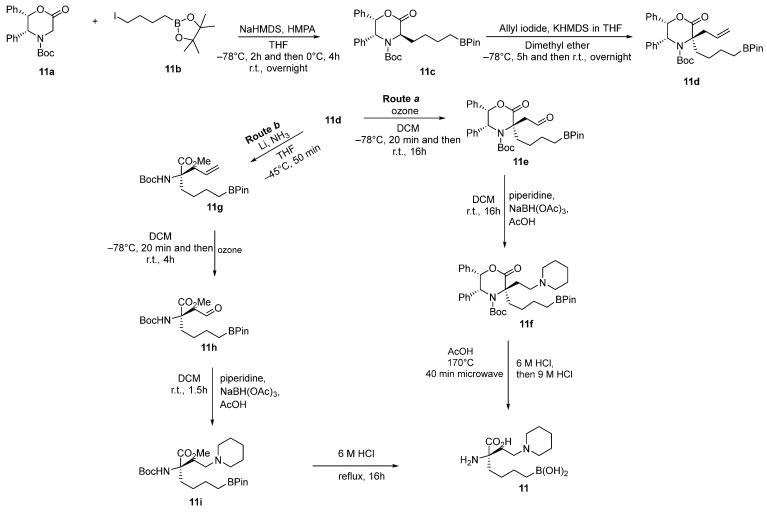
Synthesis of compound **11**.

The first second-generation molecule discussed in this review is (R)-2-amino-6-borono-2-(2-piperidin-1-yl) ethyl) hexanoic acid (**11**), which demonstrated inhibition activity against hARG-1 and hARG-2 with IC_50_ values of 223 nM and 509 nM, respectively [24]. Its cellular activity against hARG-1 in CHO cells was also confirmed (IC_50_ = 509 nM) [22]. The patented synthesis of compound **11** (Scheme 7) began with (4S,5S)-tert-butyl-6-oxo-4,5-diphenyl-1,3-oxazinane-3-carboxylate (**11a**), which was alkylated with 2-(4-iodobutyl)-4,4,5,5-tetramethyl-1,3,2-dioxaborolane (**11b**) using NaHMDS and HMPA in THF at −78 °C, forming boronate (**11c**). A second alkylation with allyl iodide, using potassium bis(trimethylsilyl)amide (KHMDS) as the base and DME (dimethoxyethane) as the solvent, yielded oxazinone **11d**, which followed two possible synthetic routes a or b. In route a, **11d** underwent ozonolysis in DCM at −78 °C (**11e**) followed by reductive amination with piperidine, NaBH(OAc)_3_, and AcOH in DCM to produce amine **11f** in a 95% yield. The auxiliary oxazinone was then removed by treatment with 6 M HCl in a microwave reactor at 170 °C for 40 min, yielding compound **11** in a 87% yield. For route b, the steps were reversed. First **11d** was treated with Li in liquid ammonia to produce (R)-methyl 2-allyl-2-(tert-butoxycarbonylamino)-6-(4,4,5,5-tetramethyl-1,3,2-dioxaborolan-2-yl)hexanoate (**11g**) as a colourless oil. This intermediate was then subjected to ozonolysis in DCM at −78 °C (**11h**), followed by reductive amination with piperidine, NaBH(OAc)_3_, and AcOH forming amine **11i** in 67% yield. The deprotection of **11i** with 6 M HCl under reflux for 16 h produced the final compound **11** as a dihydrochloride salt [51]. The two synthetic procedures for compound **11** (route a and route b) were developed to facilitate large-scale synthesis. Route a required fewer synthetic steps and provided a higher overall yield compared to route b. The need to optimize the synthesis of compound **11,** addressed by the same authors who designed this inhibitor, was driven by the need for a precise configuration of the target inhibitor and the requirement to start with a flexible intermediate, such as the aldehyde, which could be prepared on a large scale and with high enantioselectivity.

**Scheme 8:**
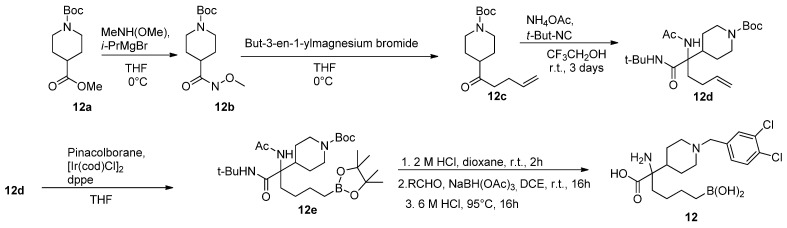
Synthesis of compound **12**.

Among α, α-disubstituted amino acid-based ARG inhibitors, compound 2-amino-6-borono-2-(1-(3,4-dichlorobenzyl) piperidin-4-yl) hexanoic acid (**12**), stands out. This derivative, featuring a piperidine ring linked to a quaternary amino acid centre, was synthesized by Golebiowski et al. (Scheme 8) [26]. It demonstrated potent inhibitory activity with IC_50_ values of 200 nM for hARG-1 and 290 nM for hARG-2 [16]. The synthesis of compound **12** began with the reaction of 1-tert-butyl-4-methyl piperidine-1,4-dicarboxylate (**12a**) with N,O-dimethylhydroxylamine and i-PrMgBr in THF, forming Weinreb amide **12b**. Next, **12b** underwent substitution with but-3-en-1-yl-magnesiumbromide in THF at 0 °C under N_2_ yielding tert-butyl 4-(pent-4-enoyl) piperidine-1-carboxylate (**12c**) in a 91% yield. In the third step, an Ugi reaction was performed by treating ketone **12c** with tert-butyl isocyanide and ammonium acetate (NH_4_OAc) in 2,2,2-trifluoroethanol, producing intermediate tert-butyl-4-(2-acetamido-1-(tert-butylamino)-1-oxohex-5-en-2-yl) piperidine-1-carboxylate (**12d**). This was followed by a hydroboration reaction using pinacolborane, chloro-1,5-cycloctadiene iridium (I) dimer ([Ir(cod)Cl]_2_), and 1,2-bis(diphenylphosphino)ethane (dppe) in THF, to yield derivative **12e**. A subsequent step included the deprotection of the piperidine ring with 2 M HCl in dioxane at r.t. for 2 h. The deprotected piperidine was then N-alkylated with 3,4-dichlorobenzaldehyde and NaBH(OAc)_3_ in 1,2 dichloroethane (DCE) at r.t. for 16 h. Final acid deprotection with 6 M HCl yielded the target compound **12** [26,51]. This synthetic procedure was optimized by the authors and involved seven steps, each achieving notably high yields. The optimization process incorporated a multi-component reaction strategy, specifically the Ugi reaction, using a ketone derivative (**12c**) as a model substrate and tert-butyl isocyanide. Optimal conditions were achieved by using ammonium acetate as both the amino and acid component, along with replacing methanol with trifluoroethanol as the solvent. This adjustment effectively suppressed the competitive Passerini reaction.

**Scheme 9:**
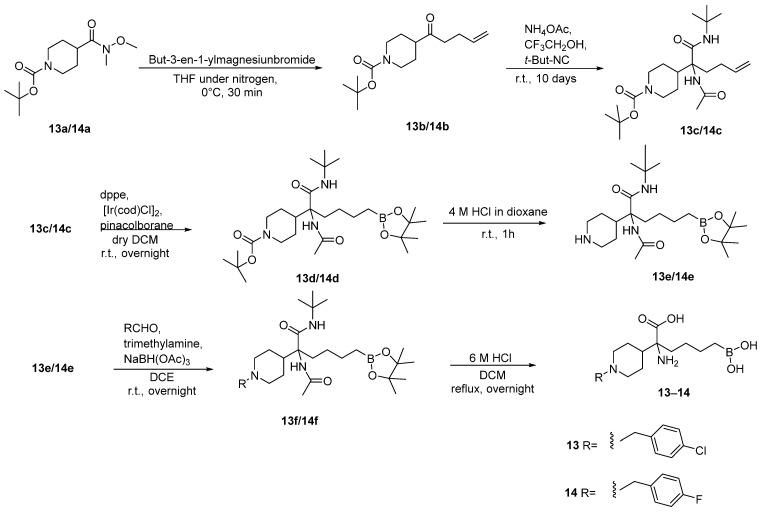
Synthesis of compounds **13** and **14**.

Two additional N-alkylated piperidine derivatives are 2-amino-6-borono-2-(1-(4-chlorobenzyl) piperidin-4-yl)-hexanoic acid (**MARS**, **13**) and 2-amino-6-borono-2-(1-(4-fluorobenzyl)piperidin-4-yl)hexanoic acid (**FMARS**, **14**), which differ in the halogen substituent. MARS exhibited IC_50_ values of 0.9 μM for hARG-1 and 0.7 μM for hARG-2, while FMARS showed IC_50_ values of 1.1 μM for hARG-1 and 0.4 μM for hARG-2 [52]. The synthesis of both compounds (Scheme 9), reported by Clemente et al., followed the patented protocol established by Adam Golebiowski et al. [51,52]. The strategy was similar to that used for compound **12**, with the key difference being the use of a reductive amination step (4-chloro and 4-fluoro benzadelhydes for MARS and FMARS, respectively).

Another class of ARG inhibitors includes tropane derivatives (**15**–**19**), characterized by a two-carbon bridge within the piperidine ring, which enhances their activity. These compounds demonstrated significant inhibition of hARG-1 and hARG-2, with IC_50_ values ranging from nanomolar to micromolar levels [22,24,26,52,53,54]. The increased potency of tropane derivatives **15**–**19** compared to the simpler piperidine derivatives **12**–**14** has been thoroughly investigated. For derivatives **12**–**14**, the piperidine ring adopts a chair conformation, with the nitrogen atom interacting with two aspartate residues in the binding pocket of ARG-1 and ARG-2 through a water molecule. In contrast, for derivatives **15**–**19**, the two-carbon bridge of the tropane forces the piperidine ring into a boat conformation, enabling the nitrogen atom to establish direct contact with one of the aspartate residues in the pocket of interest. The other aspartate residue is still contacted via the water molecule. Thus, the tropane derivatives benefit from two key advantages that explain their enhanced potency: the fixed ring geometry positions the nitrogen atom optimally (resulting in an entropy gain) and the nitrogen establishes direct contact with one aspartate residue, bypassing the need for water-mediated interaction. Furthermore, this significant improvement in in vitro activity was accompanied by reduced polarity and an improved pharmacokinetic profile.

**Scheme 10:**
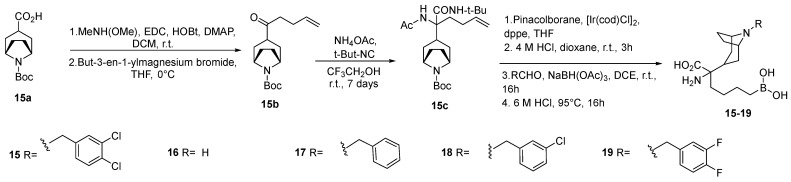
Synthesis of compounds **15**–**19**.

The synthesis of the derivatives **15**–**19** involved a seven-step protocol (Scheme 10) [55]. The initial four steps were consistent across all compounds. For the synthesis of compound 2-amino-2-(8-azabicyclo [3.2.1]octan-3-yl)-6-boronohexanoic acid (**16**), the fifth and sixth steps were omitted, proceeding directly to the final stage. For compounds 2-amino-6-borono-2-((1R,5S)-8-(3,4-dichlorobenzyl)-8-azabicyclo[3.2.1]octan-3-yl)hexanoic acid (**ABHtrop**, **15**), 2-amino-2-(8-benzyl-8-azabicyclo[3.2.1]octan-3-yl)-6-boronohexanoic acid (**17**), 2-amino-6-borono-2-(8-(3-chlorobenzyl)-8-azabicyclo[3.2.1]octan-3-yl) hexanoic acid (**18**), and 2-amino-6-borono-2-(8-(3,4-difluorobenzyl)-8-azabicyclo [3.2.1]octan-3-yl) hexanoic acid (**19**), the sixth step varied depending on the aldehyde reagent used.

The synthetic strategy for these tropane ring analogues closely resembles that developed by the same authors for compound **12**. In this case, the intermediate **15b** was used as the model substrate in the Ugi reaction. Both this intermediate and the subsequent Ugi product, **15c**, were thermodynamically favoured. Notably, the use of the trans isomer of derivative **15b** as a substrate for the Ugi reaction resulted in the same relative stereochemistry for **15c**. Starting with (1R,5S)-8-(tert-butoxycarbonyl)-8-azabicyclo[3.2.1]octane-3-carboxylic acid (**15a**), the first step was the coupling with N-methyl-N-methoxyamide using MeNH(OMe), 1-ethyl-3-(3-dimethylaminopropyl)carbodiimide (EDC), hydroxybenzotriazole (HOBt), and 4-dimethylaminopyridine (DMAP) in DCM at r.t. The intermediate underwent substitution with but-3-en-1-yl-magnesium bromide in THF at 0 °C, forming (1R,5S)-tert-butyl 3-(pent-4-enoyl)-8-azabicyclo[3.2.1]octane-8-carboxylate (**15b**). In the next step, **15b** participated in an Ugi reaction with NH_4_OAc, tert-butyl-isocyanide, and 2,2,2-trifluoroethanol at r.t. for 7 days, yielding (1R,3s,5S)-tert-butyl 3-(2-acetamido-1-(tert-butylamino)-1-oxohex-5-en-2-yl)-8-azabicyclo[3.2.1]octane-8-carboxylate (**15c**). Notably, both ketone **15b** and Ugi product **15c** were thermodynamically favoured, with the trans isomer of **15b** yielding the same stereochemistry in **15c**. Next, **15c** underwent hydroboration reaction with pinacolborane, [Ir(cod)Cl]_2_, and dppe in THF, producing a hydroborate intermediate. This was treated with 4 M HCl in dioxane at r.t. for 3 h to selectively deprotect the tropane nitrogen. N-alkylation for compounds **15** and **17**–**19** was carried out using NaBH(OAc)_3_ and various aldehydes (3,4-dichlorobenzaldehyde for compound **15**, benzaldehyde for **17**, 3-chlorobenzaldehyde for **18**, and 3,4-difluorobenzaldehyde for **19**) in DCE. The intermediates were finally deprotected with 6 M HCl at 95 °C for 16 h, producing the desired compounds.

**Scheme 11:**
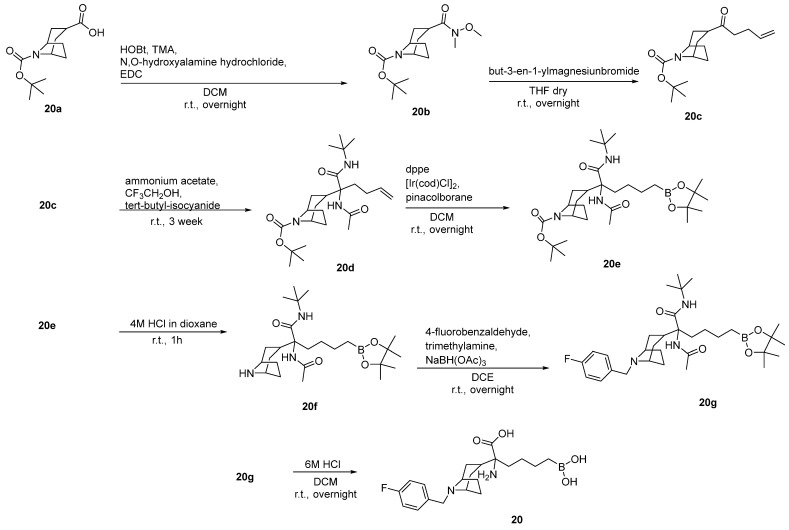
Synthesis of compound **20**.

The same strategy was applied to synthesize **FBMARS** (**20**, Scheme 11). Starting from amino acid **20a**, a coupling reaction with N,O-dimethylhydroxyalamine hydrochloride in the presence of EDC, HOBt, and trimethylamine (TMA) in DCM yielded the Weinreb amide **20b**. This intermediate was substituted with but-3-en-1-yl-magnesiumbromide in dry THF, forming ketone **20c**. Ketone (**20c**) underwent an Ugi reaction with NH_4_OAc, 2,2,2-trifluoroethanol, and tert-butyl-isocyanide, yielding **20d** after 3 weeks. Hydroboration with dppe, [Ir(cod)Cl]_2_, DCM and 4,4,5,5-tetramethyl-1,3,2-dioxaborolane produced **20e**. Subsequent treatment with 4 M HCl in dioxane for 1 h removed the tert-butyloxycarbonyl group, yielding **20f** as a salt. This salt was N-alkylated with 4-fluorobenzaldehyde in DCE using TMA and sodium triacetoxyborohydride, forming intermediate **20g**. Finally, deprotection with 6 M HCl in DCM gave **FBMARS** (**20**) [52].

**Figure 3:**
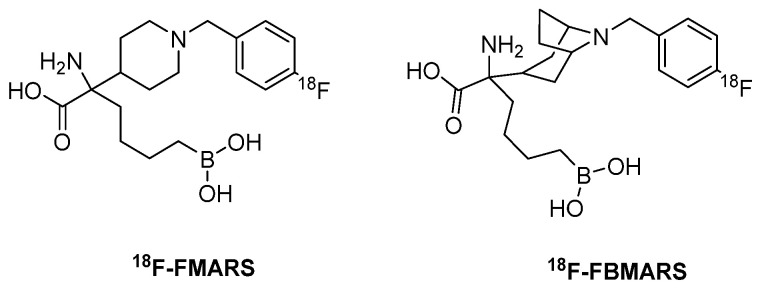
Chemical structures of compounds **^18^F-FMARS** and **^18^F-FBMARS.**

The same research group detailed the synthesis of the ^18^F-fluoroanalogues of **FMARS** (**14**) and **FBMARS** (**20**) using copper-mediated late-stage radiofluorination. These radioactive compounds are highly valuable for ARG imaging, which has proven effective in detecting and monitoring ARG-related diseases. The structures of **^18^F-FMARS** and **^18^F-FBMARS** are shown in Figure 3.

Another class of ARG inhibitors includes *N*-alkylcyclobutylamine derivatives, featuring a nitrogen atom substituted with diverse functional groups such as phenyl and biphenyl moieties, which can be further modified. As summarized in Table 1, these compounds (**21**–**46**) exhibited activity against hARG-1 and hARG-2, with potency ranging from 0.1 nM to 100 nM.

**Scheme 12:**
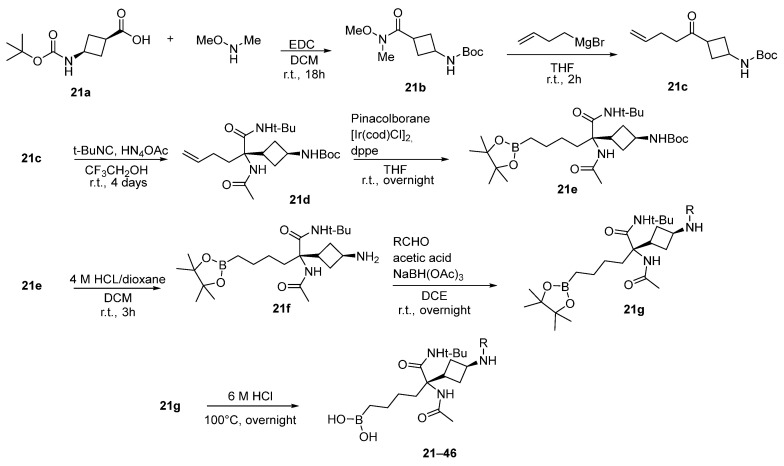
Synthesis of compounds **21**–**46**.

The synthesis of compounds **21**–**46**, patented by Van Zandt et al. and illustrated in Scheme 12, closely resembles previously described methods. The process involved seven steps differing only in the reagent used during the sixth step [55]. The first step was a coupling reaction involving (1S,3S)-3-(*tert*-butoxycarbonylamino)cyclobutanecarboxylic acid (**21a**), N,O-dimethylhydroxylamine hydrochloride, *N*-methyl-morpholine, and EDC in DCM. This reaction, carried out at r.t. over 18 h, produced *tert*-butyl(1S,3S)3-(methoxy(methyl)carbamoyl)cyclobutylcarbamate (**21b**). The second step involved the substitution of **21b** with 3-butenylmagnesium bromide in THF for 2 h at r.t., yielding *tert*-butyl-(1S,3S)-3-pent-4-enoylcyclobutylcarbamate (**21c**). In the third step, an Ugi reaction with **21c** in 2,2,2-trifluoroethanol, NH_4_OAc and *tert*-butyl isocyanide over 4 days produced *tert*-butyl(1S,3S)-3-(R-2-acetamido-1-(tert-butylamino)-1-oxohex-5-en-2-yl)cyclobutylcarbamate (**21d**). Next, **21d** underwent hydroboration with pinacolborane in the presence of [Ir(cod)Cl]_2_ and dppe in DCM, forming *tert*-butyl-(1S,3S)-3-((R)-2-acetamido-1-(tert-butylamino)-1-oxo-6-(4,4,5,5-tetramethyl-1,3,2-dioxaborolan-2-yl)hexan-2-yl)cyclobutylcarbamate (**21e**). In the fifth step, **21e** was treated with 4 M HCl in dioxane for 3 h to remove the *tert*-butyloxycarbonyl group, yielding (S)-2-acetamido-2-((1S, 3R)-3-aminocyclobutyl)-*N*-*tert*-butyl-6-(4,4,5,5-tetramethyl-1,3,2-dioxaborolan-2-yl)hexanamide (**21f**) as a hydrochloride salt. For the sixth step, **21f** was reacted overnight with acetic acid, NaBH(OAc)_3_, and various aldehydes (depending on the desired derivative) in DCE, producing the respective *N*-alkylated intermediates (**21g**). In the final step, these intermediates were treated with 6 M HCl and heated to 100 °C overnight, yielding the final products **21**–**46**, whose names are provided in Table 1.

**Scheme 13:**
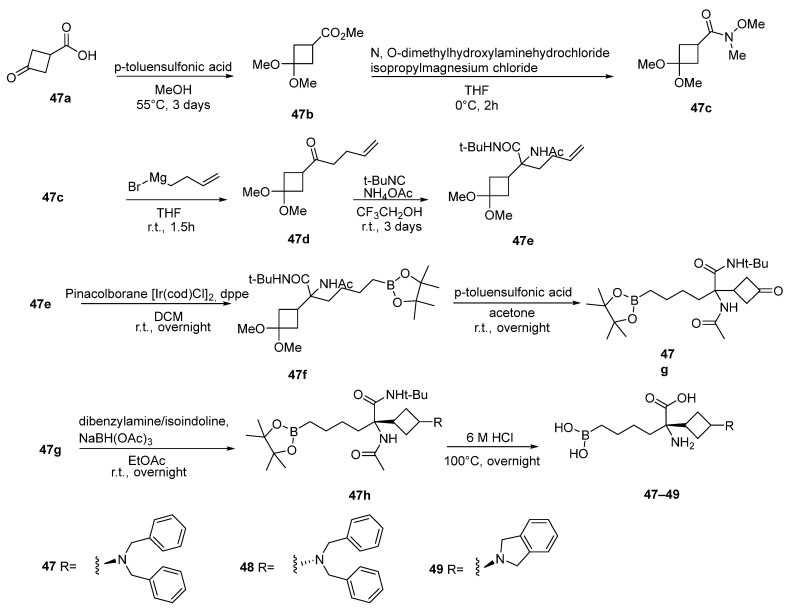
Synthesis of compounds **47**–**49**.

Further investigations into the *N*-alkylcyclobutylamine scaffold led to the development of tertiary amine derivatives (compounds **47**–**49**). However, these derivatives displayed lower potency compared to the parent compounds **21**–**46**. Their syntheses and structures are shown in Scheme 13.

The synthesis of compounds **47**–**49** followed an eight-step protocol starting with the reaction of 3-oxocyclobutanecarboxylic acid (**47a**) in MeOH and p-toluensulfonic acid at 55 °C for 3 days to yield methyl-3,3-dimethoxycyclobutanecarboxylate (**47b**). This intermediate underwent coupling with N,O-dimethylhydroxylaminehydrochloride and isopropylmagnesium chloride in THF to produce **47c**. Next, substitution with 3-butenylmagnesium bromide yielded 1-(3,3-dimethoxycyclobutyl)pent-4-en-1-one (**47d**), which was subjected to an Ugi reaction with ammonium acetate, tert-butyl-isocyanide, and 2,2,2-trifluoroethanol, producing 2-acetamido-N-tert-butyl-2-(3,3-dimethoxycyclobutyl)hex-5-enamide (**47e**). The hydroboration of **47e** with pinacolborane, [Ir(cod)Cl]_2_ and dppe generated 2-acetamido-N-tert-butyl-2-(3,3-dimethoxycyclobutyl)-6-(4,4,5,5-tetramethyl-1,3,2-dioxaborolan-2-yl)hexanamide (**47f**), which was then treated with p-toluensulfonic acid overnight in acetone to form 2-acetamido-N-tert-butyl-2-(3-oxocyclobutyl)-6-(4,4,5,5-tetramethyl-1,3,2-dioxaborolan-2-yl hexanamide (**47g**), containing an oxidized cyclobutyl ring. In the penultimate step, **47g** was reacted with either dibenzylamine (for compound **47**), its trans isomer (for **48**), or isoindoline (for **49**), followed by NaBH(OAc)_3_ reduction to form intermediates **47h**. Finally, global deprotection using 6 M HCl at 100 °C yielded the desired compounds **47**–**49** [47].

The basic side chain α-substituted ABH analogues (compounds **50**–**51**) also emerged as potent ARG inhibitors. Compound **50**, i.e., 2-amino-6-borono-2-(3-(4-(4-chlorophenyl)-4-hydroxypiperidin-1-yl)propyl)hexanoic acid, with a cyclic amine side chain, showed strong inhibitory activity (Ki < 10 nM) against both hARG-1 and 2 [22]. Compound **51**, featuring a linear amine side chain, exhibited comparable potency.

**Scheme 14:**
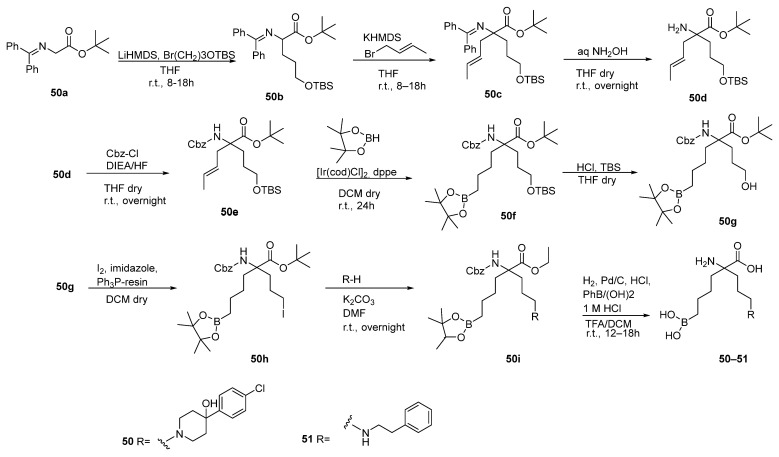
Synthesis of compounds **50**–**51**.

Their synthesis involved a nine-step protocol (Scheme 14) [56]. The initial steps consisted of sequential alkylations starting from tert-butyl-2-((diphenylmethylene)amino)acetate (**50a**) generating tert-butyl-5-(tert-butyldimethylsilyoxy)-2-(diphenylmethyleneamino)pentanoate (**50b**) and then tert-butyl-2-(3-tert-butylmethylsilyloxy)propyl)-2-(diphenylmethylenamino)hex-4-enoate (**50c**). After amine deprotection with hydroxylamine hydrochloride, the amino group was re-protected with benzyl chloroformate to yield tert-butyl-2-(benzyloxycarbonylamino)-2-(3-(tert-butidimethylsilyloxy)propyl)hex-4-enoate (**50e**). The hydroboration of **50e** with pinacolborane in the presence of [Ir(cod)Cl]_2_, and dppe in dry DCM gave tert-butyl-2-(benzyloxycarbonylamino)-2-(3-(tert-butidimethylsilyloxy)propyl)-6-(4,4,5,5-tetramethyl-1,3,2-dioxaborolan-2-yl)hexanoate (**50f**), followed by the removal of the tert-butyldimethylsilyl (TBDMS) group under acid conditions to form **50g.** The alcohol group in **50g** was iodinated to produce tert-butyl-2-(benzyloxycarbonylamino)-2-(3-iodopropyl)-6-(4,4,5,5-tetramethyl-1,3,2-dioxaborolan-2-yl)hexanoate (**50h**). In the eighth step, N nucleophilic substitution was carried out using 4-(4-chlorophenyl)piperidin-4-ol hydrochloride (for compound **50**) or 2-phenylethanamine (for compound **51**), yielding tert-butyl-2-(benzyloxycarbonylamino)-2-(3-(3-phenylpiperidin-1-yl)propyl)-6-(4,4,5,5-tetramethyl-1,3,2-dioxaborolan-2-yl)hexanoate (**50i**). Finally, global deprotection using 1 M HCl, Pd/C, TFA and DCM under argon, followed by the addition of phenyl boronic acid, afforded final products **50**–**51**.

**Scheme 15:**
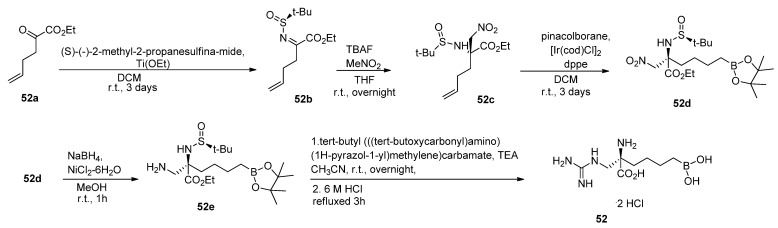
Synthesis of compound **52**.

OncoArendi Therapeutics (now Molecure) has developed (R)-2-amino-6-borono-2-(guanidinomethyl)hexanoic acid (**52**), a compound featuring a methylguanidine side chain at the α position relative to the amino acid group. The pure (R)-enantiomer demonstrated potent ARG inhibition, with an IC_50_ of 32 nM against hARG-1. The synthesis protocol of this compound is shown in Scheme 15 and is patented. The starting compound was ethyl-2-oxohex-5-enoate (**52a**), which was treated with (S)-(-)-2-methyl-2-propanesulfinamide, Ti(OEt)_4_ in DCM to form imine (**52b**), which was reacted in turn with TBAF and MeNO_2_ undergoing a nitro methylene addition on the α-carbon atom. The asymmetric, diastereoselective aza-Henry reaction led to the formation of the nitro amine ester (**52c**), which was subjected to hydroboration using pinacolborane, [Ir(cod)Cl]_2_, dppe, DCM, and the subsequent reduction of the nitro group with NaBH_4_ and NiCl_2_-6H_2_O in MeOH to obtain boronate (**52e**). The amine (**52e**) was then guanylated using *tert*-butyl(((*tert*-butoxycarbonyl)amino)(1H-pyrazol-1-yl)methylene) carbamate, TEA, and ACN and was deprotected with a solution of 6 M HCl to obtain the desired compound (**52**) [25,57].

### 3.2. Non-Basic Side Chain α-Substituted ABH Analogues

**Scheme 16:**
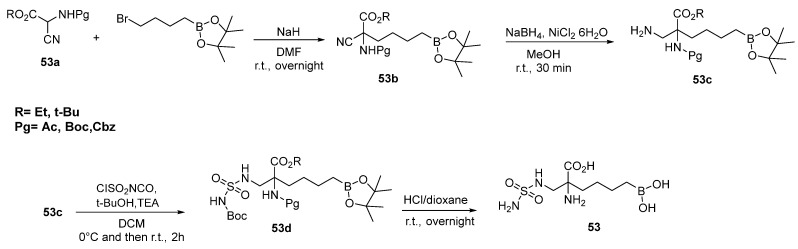
Synthesis of compound **53**.

To this subclass belong sulphamide derivatives such as 2-amino-6-borono-2-((sulfamoylamino)methyl)hexanoic acid (**53**), which exhibited an inhibition activity against hARG-1 with an IC_50_ of 330 nM [25]. The synthesis of **53** (see Scheme 16) consisted of an alkylation reaction of compound **53a** with pinacol-4-bromobutylboronate and sodium hydride (NaH) in N,N-dimethylformamide (DMF) to obtain a quaternary boronic cyanoaminoester (**53b**), which was then reduced with sodium borohydride (NaBH_4_) to form the intermediate **53c**. Compound **53c** was subsequently subjected to sulphamoylation with chlorosulfonyl isocyanate (ClSO_2_NCO) in the presence of tert-butanol in TEA and DCM at 0 °C. The final step involved acid hydrolysis with HCl/dioxane, yielding the desired boronic acid (**53**).

**Scheme 17:**
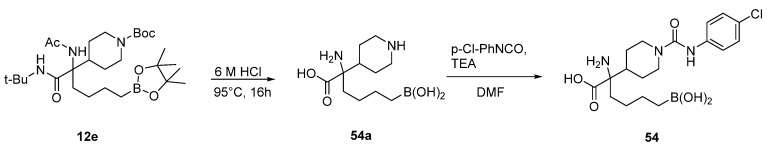
Synthesis of compound **54**.

Other compounds designed as ARG inhibitors with a non-basic side chain included derivatives **54**–**55,** which contained urea and thiourea moieties, respectively. Both **54** (2-amino-6-borono-2-(1-((4-chlorophenyl)carbamoyl)piperidin-4-yl)hexanoic acid) and **55** (2-amino-6-borono-2-(1-((4-chlorophenyl)carbamothioyl)pyrrolidin-3-yl)hexanoic acid) were capable of inhibiting hARG-1 and -2 in the range of 251 to 1000 nM. Despite their similar activities, the synthesis pathways for these compounds differed [22,51]. For compound **54** (Scheme 17), the first four-steps of its synthesis were identical to those for compound **12**, as shown in Scheme 8. Starting from intermediate **12e,** the fifth step involved an acid deprotection reaction using 6 M HCl solution at 95 °C for 16 h, giving intermediate **54a**. This intermediate was then selectively carbamylated at the piperidine nitrogen using 4-chlorophenyl isocyanate (p-Cl–PhNCO) in the presence of TEA and DMF, producing the target compound **54** [26].

**Scheme 18:**
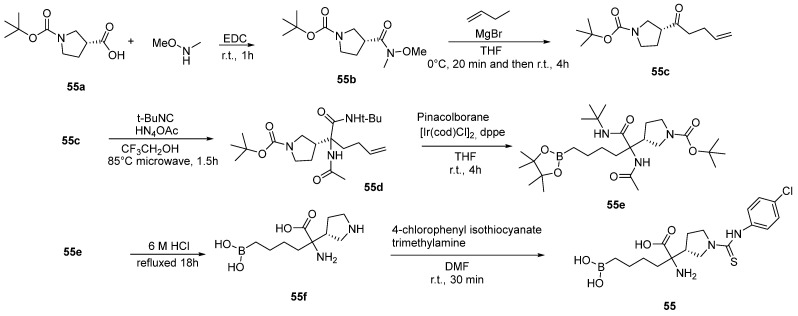
Synthesis of compound **55**.

For the synthesis of compound **55**, as shown in Scheme 18, Van Zandt et al. used **55a** as the starting material [51]. This compound underwent a coupling reaction with N,O-dimethylhydroxylamine hydrochloride and EDC, yielding intermediate **55b**, which in turn was then subjected to a substitution with 3-butenylmagnesium bromide in THF producing compound **55c**. Next, an Ugi reaction was performed with NH_4_OAc and tert-butyl isocyanide in 2,2,2-trifluoroethanol, generating derivative **55d**. This was followed by a hydroboration reaction using pinacolborane, [Ir(cod)Cl]_2_ and dppe to give tert-butyl-(3R)-3-[1-acetamido-1-(tert-butyl-carbamoyl)-5-(4,4,5,5-tetramethyl-1,3,2-dioxaborolan-2-yl)pentyl]pyrrolidine-1-carboxylate (**55e**). In the fifth step, a solution of 6 M HCl in 1,4-dioxane was added to **55e**, yielding intermediate **55f**, which was then treated with 4-chlorophenyl-isothiocyanate in TEA and DMF to obtain the desired compound **55**. In addition to the inhibitors mentioned, several other compounds featuring different substituents in their side chains were synthesized, through their activities were found to be comparatively lower [58].

Comparing the two subclasses of second-generation ARG inhibitors, it was observed that the presence of a basic side chain in the α-substituted ABH derivatives led to a notable improvement not only in activity but also in selectivity against both ARG-1 and ARG-2, compared to their non-basic counterparts. This enhancement was attributed to the formation of an additional hydrogen bond, mediated by a water molecule, between the basic centre in the side chain of the ARG inhibitor and an aspartate residue in the pocket of both ARG-1 and ARG-2—a feature absent in non-basic side chain α-substituted ABH analogues. However, despite the improved selectivity and activity, the presence of a basic side chain did not result in significant improvements in bioavailability compared to non-basic analogues, and the pharmacokinetic profile still required optimization.

## 4. Third-Generation Inhibitors

**Scheme 19:**
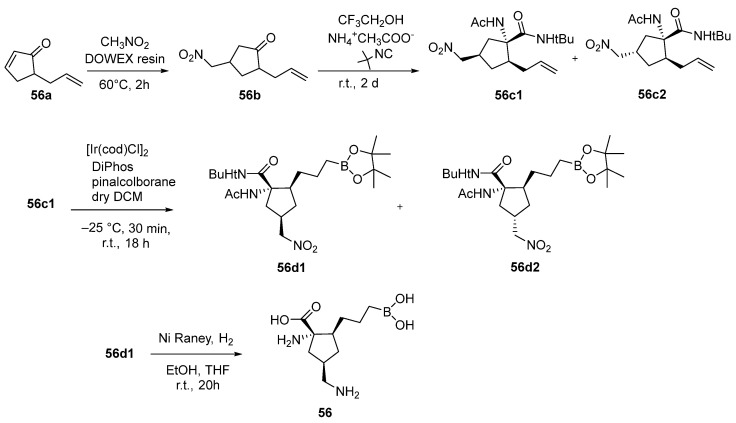
Synthesis of compound **56**.

The third generation of inhibitors features ring-constrained cyclic ABH analogues, including compounds based on modified cyclopentyl and pyrrolidine structures. These compounds have an alkyl linker with a boronic acid group inserted at ring position 2. One example is (1S,2S,4R)-1-amino-4-(aminomethyl)-2-(3-boronopropyl)cyclopentanecarboxylic acid (**56**), which showed an IC_50_ of 0.1–0.250 nM for both hARG-1 and hARG-2 in a colorimetric assay. While the cited patent describes many analogues of **56,** the lack of its further development is likely due to its unsatisfactory pharmacokinetic properties. These issues could potentially be addressed through strategies such as the prodrug approach or the design of nanodelivery systems, which, as demonstrated in thousands of studies, have been shown to overcome various pharmacokinetic and related limitations. Compound **56** was synthesized by Van Zandt et al. according to the Scheme 19 [59].

The synthesis began with commercially available 5-(propene-3-yl)cyclopent-2-enone (**56a**), which was treated in nitromethane with the DOWEX^®^ 550A-OH resin at 60 °C to obtain the nitroderivative (**56b**). This was then dissolved in 2,2,2-trifluoroethanol and reacted with NH_4_OAc and tert-butylisonitrile under N_2_ for 2 days, affording two isomers, **56c1** and **56c2**, with acetamino and allyl substituents in the syn-relative position. Isomer **56c1** was stirred in DCM and treated with pinacolborane, [Ir(cod)Cl]_2_ and Diphos^®^ at –25 °C, yielding **56d** (isomers **56d1** and **56d2**). For the final step, a solution of (1S,2S,4R)-1 -acetamido-N-tert-butyl-4-(nitromethyl)-2-(3-(4,4,5,5-tetramethyl-l,3,2-dioxaborolan-2-yl)propyl)cyclopentanecarboxamide (isomer **56d1**) in ethanol and THF under N_2_ was treated with Raney nickel. The mixture was purged with H_2_ and stirred for 20 h. After purging with nitrogen, the mixture was filtered through Celite^®^, and the filtrate concentrated under reduced pressure. The crude product was dissolved in HCl/acetic acid/H_2_O in a pressure bottle and stirred for 2 h at 60 °C, then capped and heated for 18 h at 130 °C. After cooling to r.t. and uncapping, the desired compound **56** was obtained after chromatography purification as a white foam.

**Figure 4:**
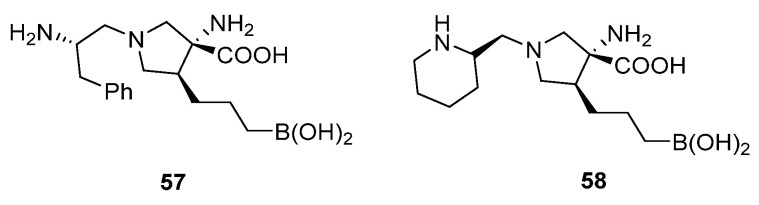
Chemical structures of compounds **57**–**58**.

A wide range of pyrrolidine-derived compounds has been developed through further optimization. The ring-constrained pyrrolidines help reduce entropy by positioning the quaternary amino acid moiety in an optimal orientation for binding. Notably, the presence of a cyclopentane derivative forces the amino group and boronic acid side chain into an anti orientation, preventing van der Waals interactions with active site residues. Furthermore, the atoms within the ring act as scaffolds for incorporating additional substituents that can form hydrogen bonds with the aspartate residues in the ARG-1 and ARG-2 pockets. These structural features collectively contribute to the enhanced potency of these compounds. The most effective compound in this series was **NED-3228** (**57**, Figure 4), which showed IC_50_ values for hARG-1 and hARG-2 of 1.3 and 8.1 nM, respectively. Another notable compound, **58** (see Figure 4), features an N-(piperidin-2-yl-methyl) substituent in place of the N-(2-amino-3-phenylpropyl) group in **57**. This compound exhibited IC_50_ values for hARG-1 and hARG-2 of 2.6 and 14 nM, respectively.

**Scheme 20:**
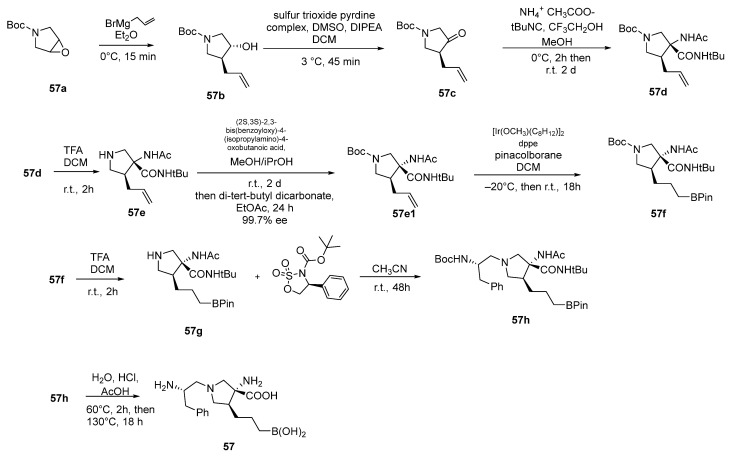
Synthesis of compound **57**.

Compound **57** was synthesized by Van Zandt et al. through a nine-step process, as outlined in Scheme 20 [60]. The synthesis began by reacting a solution of tert-butyl-6-oxa-3-azabicyclo[3.1.0]hexane-3-carboxylate (**57a**) in dry Et_2_O with allyl magnesium bromide at 0 °C. After stirring for 15 min, the reaction produced tert-butyl-trans-3-allyl-4-hydroxypyrrolidine-1-carboxylate (**57b**). This was then treated with sulphur trioxide pyridine complex in DMSO under nitrogen, yielding ketone intermediate **57c**. Compound **57c** was reacted with tert-butylisocyanide and ammonium acetate, resulting in a mixture of anti- and syn-isomers of **57d**. Next, the BOC group was removed using DCM and TFA giving racemic **57e.** To obtain the pure enantiomer **57e1**, racemic **57e** was treated with (2S,3S)-2,3-bis(benzoyloxy)-4-(isopropylamino)-4-oxobutanoic acid in warm MeOH/i-PrOH. After cooling, the desired salt crystallized out, and the crystals were filtered, washed, and dried to give **57e1** with 99.7% ee. In the following step, **57e1** was reacted with pinacolborane in the presence of [Ir(cod)Cl]_2_ and dppe in DCM to afford **57f** as a white solid. A BOC deprotection with an excess of TFA in dry DCM under N_2_ yielded **57g**. This was then reacted with tert-butyl-(S)-4-phenyl-1,2,3-oxathiazolidine-3-carboxylate 2,2-dioxide in ACN at r.t. for 2 days, producing **57h**, which was used directly without further purification. Finally, **57h** was deprotected in glacial acetic acid, water, and concentrated hydrochloric acid in a pressure bottle. After stirring at 60 °C for 2 h, the mixture was capped and heated to 130 °C for 18 h to yield **NED-3228** (compound **57**) as a white solid.

**Scheme 21:**
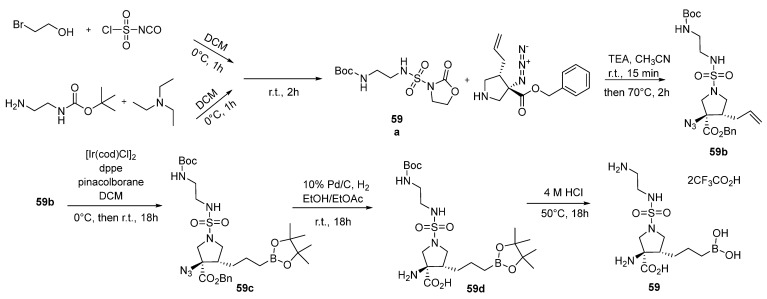
Synthesis of compound **59**.

Compound (3R,4S)-3-amino-1-(N-(2-aminoethyl)sulfamoyl)-4-(3-boronopropyl)pyrrolidine-3-carboxylic acid (**59**) also demonstrated strong activity against hARG-1 and hARG-2, with IC_50_ values ranging from 0.1 to 100 nM for both enzymes. Compound **59** was synthesized by Wan et al. in 2018, following the procedure outlined in Scheme 21 [61]. The synthesis began with the reaction between sulfurisocyanatidic chloride and 2-bromoethan-1-ol in DCM, followed by the addition of tert-butyl-(2-aminoethyl)carbamate and TEA in DCM. The intermediate (tert-butyl (2-((2-oxooxazolidine)-3-sulfonamido)ethyl)carbamate) **59a** was reacted with a solution of (rac)benzyl trans-4-allyl-3-azidopyrrolidine-3-carboxylate in ACN and TEA, resulting in **59b** ((rac)(benzyl trans-(4-allyl-3-azido-1-(N-(2-((tert-butoxycarbonyl)amino)ethyl)sulfamoyl)pyrrolidine-3-carboxylate). Next, **59b** was hydroborated with pinacolborane, [Ir(cod)Cl]_2_ and dppe in DCM producing **59c** ((rac)(benzyl trans-3-azido-1-(N-(2-((tert-butoxycarbonyl)amino)ethyl)sulfamoyl)-4-(3-(4,4,5,5-tetramethyl-1,3,2-dioxaborolan-2-yl)propyl)pyrrolidine-3-carboxylate)). Compound **59c** was catalytically hydrogenated in a mixture of EtOAc/EtOH giving rise to **59d** ((rac)-trans-3-amino-1-(N-(2-((tert-butoxycarbonyl)amino)ethyl)sulfamoyl)-4-(3-(4,4,5,5-tetramethyl-1,3,2-dioxaborolan-2-yl)propyl)pyrrolidine-3-carboxylic acid). Finally, **59d** was deprotected in HCl at 50° C. This reaction yielded the desired compound, (rac)-3-amino-1-(N-(2-aminoethyl)sulfamoyl)-4-(3-boronopropyl)pyrrolidine-3-carboxylic acid (**59**) that was purified by HPLC and isolated as a 1:2 trifluoroacetic acid salt.

**Scheme 22:**
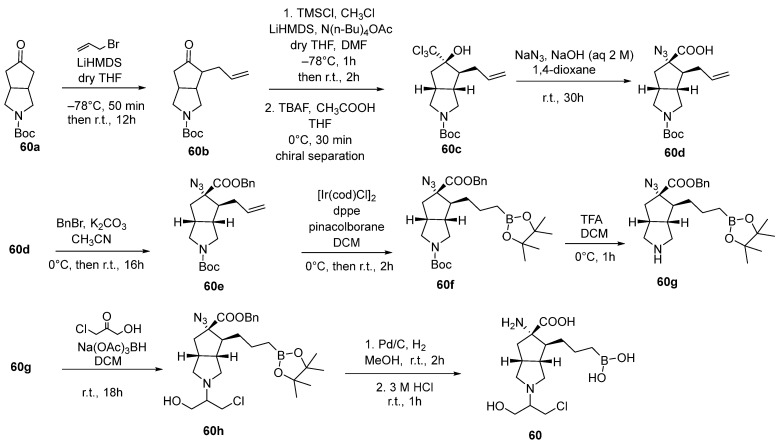
Synthesis of compound **60**.

The bicyclic inhibitors (3aR,4S,5S,6aR)-5-amino-4-(3-boronopropyl)-2-(1-chloro-3-hydroxypropan-2-yl)octahydrocyclopenta[c]pyrrole-5-carboxylic acid (**60**), (1S,2S,3aR,4S,5S,6aS)-2-amino-1-(3-boronopropyl)-4-fluoro-5-(methylamino)octahydropentalene-2-carboxylic acid (**61**), and (5S,7S,8S)-7-amino-8-(3-boronopropyl)-1-azaspiro[4.4]nonane-7-carboxylic acid (**62**) represent a distinct class of inhibitors, independently developed by Merck Sharp & Dohme Corp. and Arcus Biosciences Inc. These compounds are derivatives of octahydrocyclopenta[c]pyrrole with various nitrogen atom substituents. Foley et al. synthesized a range of bicyclic boronic acid derivatives, with compound **60** proving to be the most promising, showing an IC_50_ < 100 nm against hARG-1. Compound **61** demonstrated an IC_50_ of 6.9 nM against hARG-1 performed by Thioornithine Generating Assay (TOGA). The general synthesis scheme for compound **60** is shown in Scheme 22 [62].

In the first step, tert-butyl-5-oxohexahydrocyclopenta[c]pyrrole-2(1H)-carboxylate (**60a**) was alkylated under the action of allyl bromide and LiHMDS in THF. This produced the allylated compound tert-butyl 4-allyl-5-oxohexahydrocyclopenta[c]pyrrole-2(1H)-carboxylate (**60b**). Subsequently, a reaction of ketone **60b** with CHCl_3_ and chlorotrimethylsilane in strongly basic conditions, followed by the deprotection of trimethylsilyl group under the action of acetic acid and tetrabutylammonium fluoride in THF gave rise to the racemic trichloromethyl derivative **60c**. Chiral column chromatography was used to isolate the desired enantiomer. The treatment of **60c** with NaN_3_ and NaOH produced the crude carboxilic acid **60d** ((3aR,4S,5S,6aR)-4-allyl-5-azido-2-(tert-butoxycarbonyl)octahydrocyclopenta[c]pyrrole-5-carboxylic acid). The carboxylic group was protected as benzylic ester using benzyl bromide and K_2_CO_3_ in dry ACN. Next, **60e** was subjected to a hydroboration reaction with [Ir(cod)Cl]_2_, dppe, and pinacolborane in DCM. After purification, the product 5-benzyl 2-(tert-butyl) (3aR,4S,5S,6aR)-5-azido-4-(3-(4,4,5,5-tetramethyl-1,3,2-dioxaborolan-2-yl)propyl)hexahydrocyclopenta[c]pyrrole-2,5(1H)-dicarboxylate (**60f**) was obtained. The BOC group was removed by treating **60f** with DCM and TFA, giving the free amine (**60g**) in a 99% yield. Amine (**60g**) in DCM was reacted with 1-chloro-3-hydroxy acetone and Na(OAc)_3_BH, yielding benzyl (3aR,4S,5S,6aR)-5-azido-2-(1-chloro-3-hydroxypropan-2-yl)-4-(3-(4,4,5,5-tetramethyl-1,3,2-dioxaborolan-2-yl)propyl)octahydrocyclopenta[c]pyrrole-5-carboxylate (**60h**), which was used without purification. Finally, **60** was obtained by the hydrogenation of **60h** with 10% Pd/C in MeOH and deprotection with 3M HCl at r.t. for 1 h. Purification by reverse phase C_18_ chromatography yielded the target compound **60** as a white solid.

**Scheme 23:**
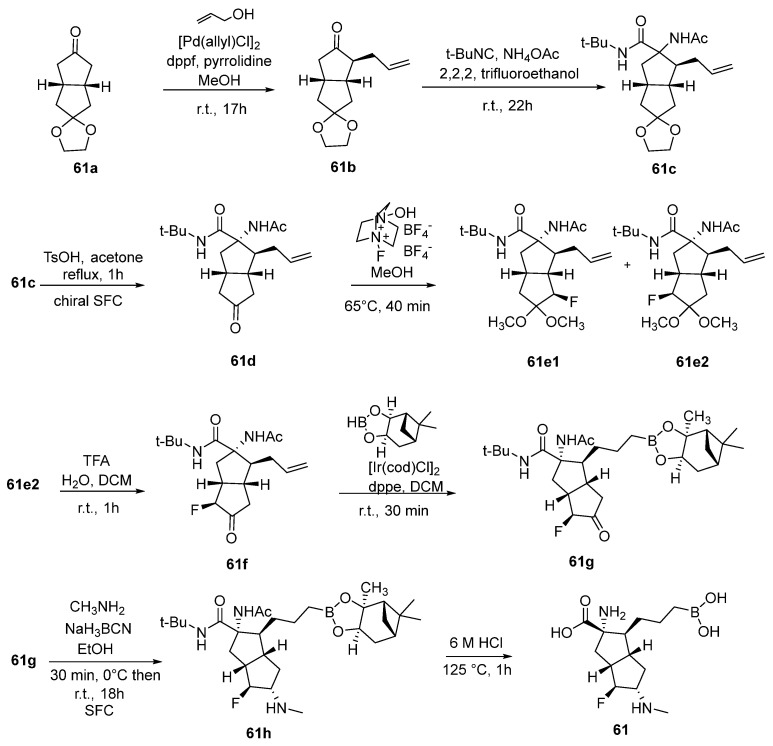
Synthesis of compound **61**.

The synthesis of **61** (Scheme 23) began by the introduction of allyl group into the 4-position of (3αR,6αS)-tetrahydro-1H-spiro[pentalene-2,2′-[1,3]dioxolan]-5(3H)-one (**61a**) under the action of allyl alcohol, 1,1′-ferrocenediyl-bis(diphenylphosphine) (dppf), and allylpalladium(II) chloride dimer in MeOH, producing **61b**. To obtain **61c**, an Ugi reaction with (3aR,4S,6aS)-4-allyltetrahydro-1H-spiro[pentalene-2,2′-[1,3]dioxolan]-5(3H)-one (**61b**), NH_4_OAc, and tert-butyl isocyanide in 2,2,2-trifluoroethanol was performed. (3αR,4S,6αS)-5-acetamido-4-allyl-N-(tert-butyl)hexahydro-1H-spiro[pentalene-2,2′-[1,3]dioxolane]-5-carboxamide (**61c**) was used without further purification. The deprotection of the dioxalan moiety with p-toluenesulfonic acid in acetone yielded a racemic mixture of epimers (~1:1) resolved using chiral supercritical-fluid chromatography (SFC) to isolate **61d**. For the next step, (1S,2S,3aS,6aR)-2-acetamido-1-allyl-N-(tert-butyl)-5-oxooctahydropentalene-2-carboxamide (**61d**), was reacted with 1-fluoro-4-hydroxy-1,4-diazoniabicyclo[2,2,2]octane bis(tetrafluoroborate) in MeOH at 65 °C for 40 min. This reaction gave two products: (1S,2S,3aS,6R,6aR)-2-acetamido-1-allyl-N-(tert-butyl)-6-fluoro-5,5-dimethoxyoctahydropent alene-2-carboxamide (**61e1**) and (1S,2S,3aR,4S,6aS)-2-acetamido-1-allyl-N-(tert-butyl)-4-fluoro-5,5-dimethoxyoctahydropentalene-2-carboxamide (**61e2**). Treating **61e2** with H_2_O and TFA in DCM at 23 °C and stirring for 1 h provided 1S,2S,3aS,6R,6aR)-2-acetamido-1-allyl-N-(tert-butyl)-6-fluoro-5-oxooctahydropentalene-2-carboxamide (**61f**) as a white solid. The hydroboration of **61f** was performed by adding it in DCM to a solution of (+)-pinanediolborane, [Ir(cod)Cl]_2_, and dppe under N_2_ at 23 °C. Stirring for 20 min yielded crude (1S,2S,3aS,6R,6aR)-2-acetamido-N-(tert-butyl)-6-fluoro-5-oxo-1-(3-((3aS,4S,6S,7aR)-3a,5,5-trimethylhexahydro-4,6-methanobenzo[d][1,3,2]dioxaborol-2yl)propyl)octahydropentalene-2-carboxamide (**61g**), used in the next step without further purification. The reaction of **61g** with methylamine in absolute EtOH at 0 °C, followed by sodium cyanoborohydride, produced **61h**. Finally, the deprotection of **61h** using 6 M HCl provided the final product **61** as a white solid [63].

Bicyclic systems, like **62**, demonstrated strong activity against both hARG-1 and hARG-2. Compound **62** inhibited hARG-1 with an IC_50_ of 2.1 nM. However, comparing its activity to other compounds is challenging because Merck utilized the (TOGA), while other groups used a colorimetric assay based on ARG-induced urea production.

**Scheme 24:**
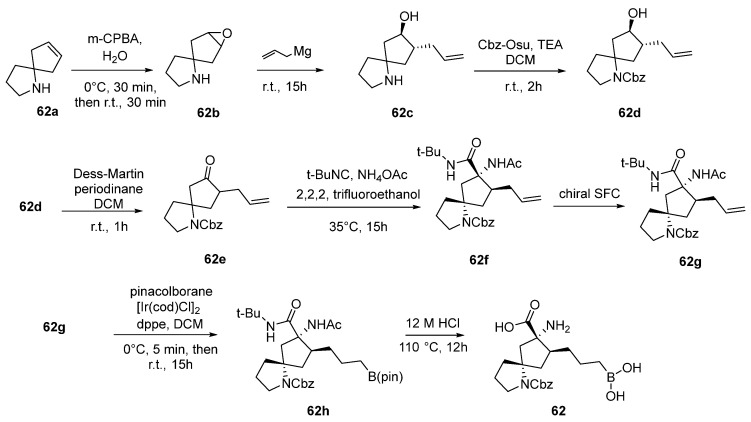
Synthesis of compound **62**.

The synthesis of **62** (Scheme 24), as reported by Mitcheltree et al., started with the oxidation of 1-azaspiro[4.4]non-7-ene (**62a**) to produce crude **62b**, which was used without purification. Reacting 6-oxaspiro[bicyclo[3.1.0]hexane-3,2′-pyrrolidine (**62b**) with allylmagnesium bromide in Et_2_O at 0 °C under N_2_ gas for 15 h yielded (7R,8R)-8-allyl-1-azaspiro[4.4]nonan-7-ol (**62c**) as a pale-yellow oil. This intermediate was also used directly. Next, TEA and benzyl (2,5-dioxopyrrolidin-1-yl) carbonate (Cbz-OSu) were added to **62c** in DCM, stirred for 2 h at r.t. and yielded (7R,8R)-7-allyl-8-hydroxy-1-azaspiro[4.4]nonane-1-carboxylate (**62d**) as a colourless oil. Oxidizing **62d** with Dess–Martin periodinane provided benzyl 7-allyl-8-oxo-1-azaspiro[4.4]nonane-1-carboxylate (**62e**), also a colourless oil. The subsequent reaction of **62e** with NH_4_OAc and tert-butyl isocyanide in 2,2,2-trifluoroethanol at 35 °C for 15 h produced benzyl (5S,7S,8S)-7-acetamido-8-allyl-7-(tert-butylcarbamoyl)-1-azaspiro[4.4] nonane-1-carboxylate (**62f**), a racemic mixture. Chiral separation using SFC gave the enantiomer (5S,7S,8S)-7-acetamido-8-allyl-7-(tert-butylcarbamoyl)-1-azaspiro[4.4]nonane-1-carboxylate (**62g**) as a viscous oil. For further modification, **62g** was reacted with dppe, [Ir(cod)Cl]_2_, and pinacolborane in DCM under inert conditions, yielding (5S,7S,8S)-7-acetamido-7-(tert-butylcarbamoyl)-8-(3-(4,4,5,5-tetramethyl-1,3,2-dioxaborolan-2-yl)propyl)-1-azaspiro[4.4]nonane-1-carboxylate (**62h**) as a viscous semisolid. Finally, the deprotection of **62h** in 12 M HCl at 110 °C for 12 h produced the target compound (5S,7S,8S)-7-amino-8-(3-boronopropyl)-1-azaspiro[4.4]nonane-7-carboxylic acid (**62**) as a viscous oil [63].

Highly effective inhibitors of ARG activity include 2-substituted alkylamines, such as the compound (1R,2S,5R)-1-acetamido-5-(2-boronoethyl)-2-(piperidin-1-ylmethyl)cyclohexanecarboxylic acid (**63**). This compound exhibited potent inhibitory activity, with IC_50_ values for hARG-1 and hARG-2, ranging from 1 to 100 nM and 100 to 1000 nM, respectively. In cellular assays, compound **63** demonstrated notable activity with an IC_50_ of up to 100 nM. Other derivatives, like compound **64** (featuring a hydroxy group at position 2), were less potent than the piperidine analogue **63**. Compound **64** showed intracellular activity between 10 and 100 μM and IC_50_ < 1000 nM for both ARGs.

**Scheme 25:**
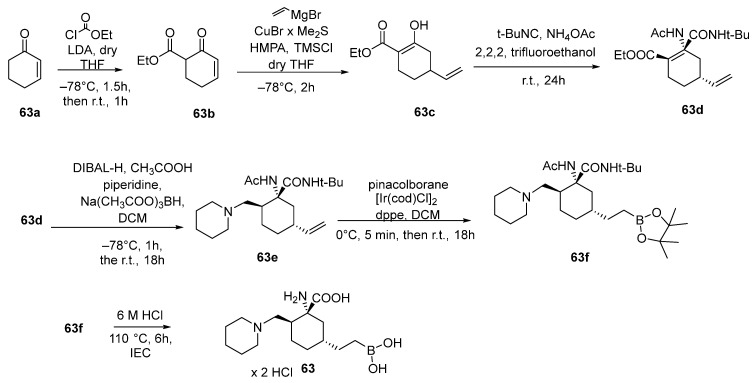
Synthesis of compound **63**.

The synthesis of **63** is outlined in Scheme 25 and began with the preparation of ethyl 2-oxocyclohex-3-enecarboxylate (**63b**) via the acylation of 2-cyclohexen-1-one (**63a**) with ethyl chloroformate under strongly basic conditions (LDA in THF). Next, ethyl 2-hydroxy-4-vinylcyclohex-1-ene-1-carboxylate (**63c**) was synthesized by reacting **63b** with vinylmagnesium bromide and CuBr x Me_2_S in the presence of TMSCl under argon at −78 °C. The treatment of **63c** with NH_4_OAc and tert-butyl isocyanide in 2,2,2 trifluoroethanol produced **63d** in a 56% yield. The subsequent reduction of ethyl rac-(1R,2R,4R)-2-acetamido-2-(tert- butylcarbamoyl)-4-vinylcyclohexane-1-carboxylate (**63d**) with diisobutylaluminium hydride (DIBAL-H) at −78 °C, followed by treatment with piperidine and sodium triacetoxyborohydride produced rac-(1R,2S,5R)-1-acetamido-N-tert-butyl)-2-(piperidin-1-yl-methyl)-5-vinylcyclohexanecarboxamide (**63e**) as a single diastereoisomer. The boronation of **63e** using pinacolborane, dppe, and [Ir(cod)Cl]_2_ in DCM at r.t. formed (1R,2S,5R)-1-acetamido-N-(tert-butyl)-2-(piperidin-1-ylmethyl)-5-(2-(4,4,5,5-tetramethyl-1,3,2-dioxaborolan-2-yl)ethyl)cyclohexanecarboxamide (**63f**). Finally, refluxing **63f** with 6 M HCl for 6h produced compound **63** as a white solid [64].

**Scheme 26:**
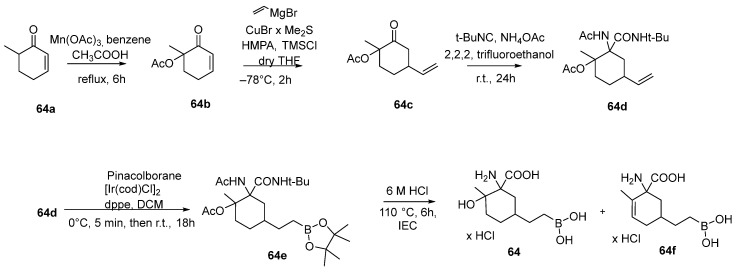
Synthesis of compound **64**.

Compound **64** was synthesized similarly to **63**, as shown in Scheme 26. Refluxing a mixture of 6-methyl-2-cyclohexenone (**64a**)^,^ benzene, acetic acid, and manganese(III) acetate dihydrate for 6 h produced **64b**. Subsequently, 1-methyl-2-oxo-4-vinylcyclohexyl acetate (**64c**) was synthesized via the 1,4-addition of vinylmagnesium bromide, catalysed by CuBr x Me_2_S in THF. Next, the Ugi reaction of **64c** with NH_4_OAc and tert-butyl isocyanide in 2,2,2 trifluoroethanol produced 2-acetamido-2-(tert-butylcarbamoyl)-1-methyl-4-vinylcyclohexyl acetate (**64d**). The hydroboronation of **64d** using pinacolborane, dppe, and [Ir(cod)Cl]_2_ in DCM at r.t. gave **64e** as a single diastereoisomer. Finally, refluxing **64e** with 6M HCl yielded 1-amino-5-(2-boronoethyl)-2-hydroxy-2-methylcyclohexanecarboxylic acid hydrochloride (**64**) as a single diastereoisomer (white solid). A by-product of **64f** was also obtained as a single diastereosomer [65].

**Scheme 27:**
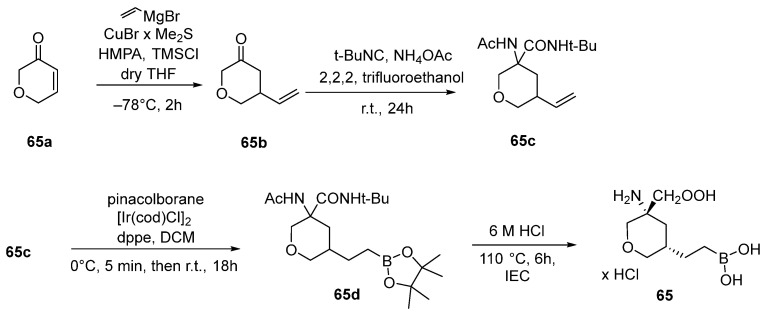
Synthesis of compound **65**.

Compound **65** was synthesized similarly to compound **64**, but with a different starting material, as illustrated in Scheme 27. The process is not described in detail. After purification via flash chromatography on DOWEX^®^ ion exchange resin, the target product, rac-(3R,5R)-3-aminno-5-(2-boronoethyl)tetrahydro-2H-pyran-3-carboxylic acid hydrochloride (**65**), was obtained as a single diastereoisomer in a 56% yield (white solid). Compound **65** demonstrated activity against both hARG-1 (IC_50_ = 100–1000 nM) and hARG-2 (IC_50_ = 1–10 mM).

**Scheme 28:**
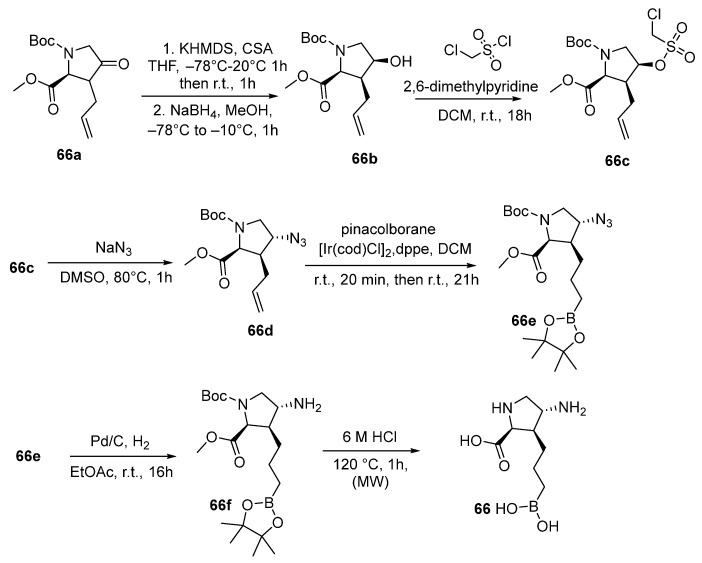
Synthesis of compound **66**.

**Scheme 29:**
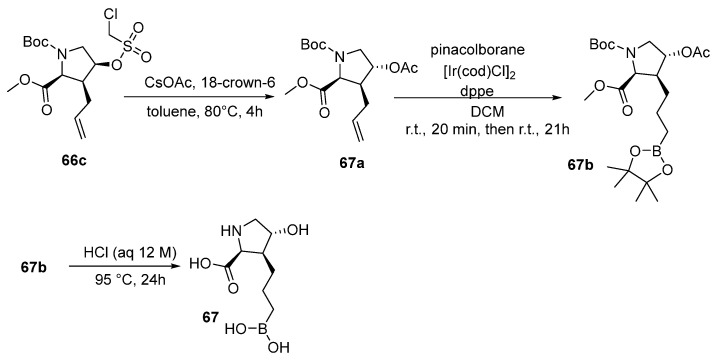
Synthesis of compound **67**.

**Scheme 30:**
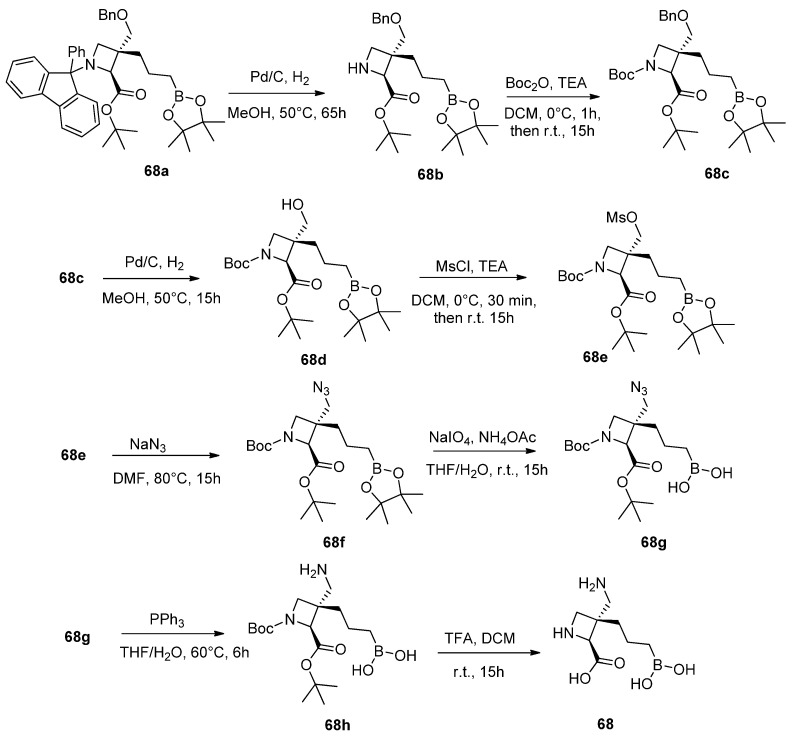
Synthesis of compound **68**.

**Scheme 31:**
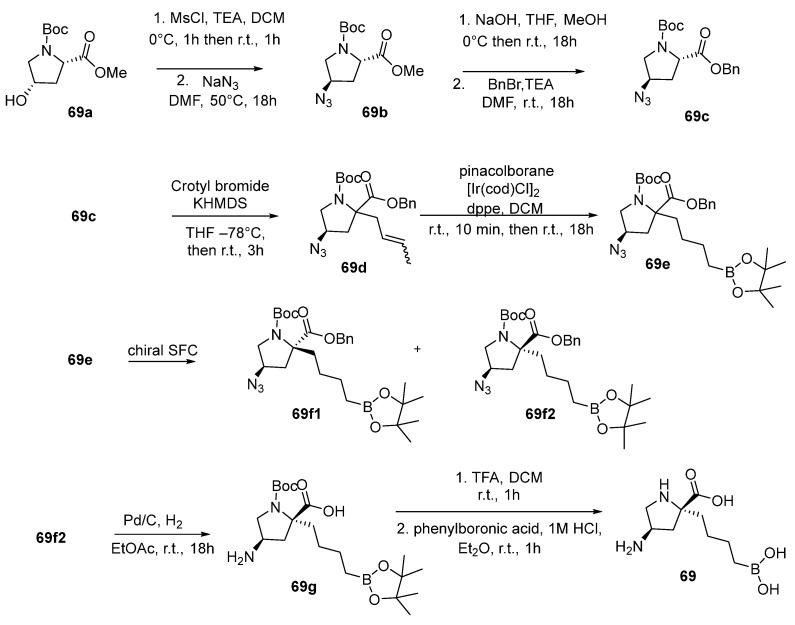
Synthesis of compound **69**.

**Scheme 32:**
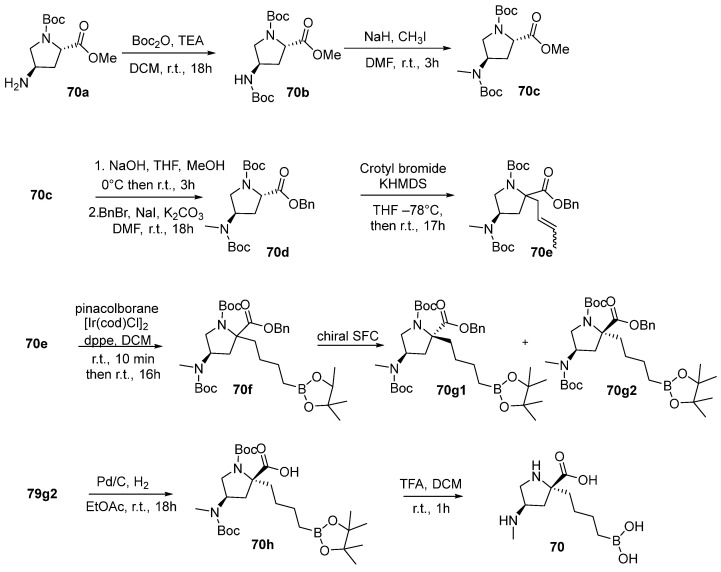
Synthesis of compound **70**.

A novel class of ARG inhibitors with a proline scaffold was developed by Sichuan Kelun-Biotech Biopharmaceutical, AstraZeneca, and Merck Sharp & Dohme. These inhibitors include proline derivatives with a boronic acid group either in position 1 (alongside a carboxyl group) or at position 2 (as in Merck’s compounds). Merck introduced compounds with various substitutions, such as hydroxyl, alkyl, piperidine, pyrrolidine, and aliphatic amino groups. Among these, a standout was the compound (2S,3R,4R)-4-amino-3-(3-boronopropyl)pyrrolidine-2-carboxylic acid (**66**), which had an amino group at position 4. This compound demonstrated significant inhibitory activity with an IC_50_ of 3.2 nM (TOGA) for hARG-1. Another notable compound, (2S,3S,4R)-3-(3-boronopropyl)-4-hydroxypyrrolidine-2-carboxylic acid (**67**), featured a hydroxyl group at position 4, arranged trans to carboxylic acid and the propylboronic acid linker. Its activity against hARG-1 was slightly lower, with an IC_50_ of 6 nM. An azetidine-based derivative, (2S,3S)-3-(aminomethyl)-3-(3-boronopropyl)azetidine-2-carboxylic acid (**68**), was the most effective homolog, showing an IC_50_ of 8 nM for hARG-1. AstraZeneca and Kelun also contributed to this class of inhibitors. AstraZeneca reported derivatives like (2R,4R)-4-amino-2-(4-boronobutyl)pyrrolidine-2-carboxilic acid (**69**), which showed IC_50_ values of 10 nM for hARG-1 and 20 nM for hARG-2. Its methyl analogue, (2R,4R)-2-(4-boronobutyl)-4-(methylamino)pyrrolidine-2-carboxylic acid (**70**), displayed even greater potency, with IC_50_ values of 3 nM for hARG-1 and 10 nM for hARG-2. Kelun’s derivatives included proline analogues substituted with hydroxyl or amino groups. Among these, compound **69** showed moderate activity in a standard assay, with an IC_50_ activity of 4.3 μM for hARG-1. The synthesis of compounds **66**–**70** follows detailed procedures outlined in Scheme 28, Scheme 29, Scheme 30, Scheme 31 and Scheme 32.

Compound **66** was synthesized as described by Achab et al. (Scheme 28) [66]. The process began with the addition of KHMDS to a solution of 1-(tertbutyl)-2-methyl (2S)-3-allyl-4-oxopyrrolidine-1,2-dicarboxylate (**66a**) in THF at –78 °C under N_2_. After stirring and warming to –20 °C, CSA was added, followed by pre-cooled MeOH and NaBH_4_ at –78 °C. Purification through silica gel chromatography yielded 1-(tert-butyl)2-methyl (2S,3S,4S)-3-allyl-4-hydroxypyrrolidine-1,2-dicarboxylate (**66b**) as a black oil. In the next step, **66b** was reacted with 2,6-dimethylpyridine and chloromethanesulfonyl chloride in DCM at 0 °C, producing crude 1-(tert-butyl) 2-methyl(2S,3S,4S)-3-allyl-4-(((chloromethyl)sulfonyl)oxy)pyrrolidine-1,2-dicarboxylate (**66c**). Without further purification, **66c** was treated with sodium azide in DMSO at 80 °C, yielding crude 1-(tert-butyl) 2-methyl (2S,3S,4R)-3-allyl-4-azidopyrrolidine-1,2-dicarboxylate (**66d**). Next, pinacolborane, [Ir(cod)Cl]_2_ and dppe were used in DCM under argon to convert **66d** into 1-(tert-butyl) 2-methyl (2S,3S,4R)-4-azido-3-(3-(4,4,5,5-tetramethyl-1,3,2-dioxaborolan-2-yl)propyl)pyrrolidine-1,2-dicarboxylate (**66e**) as a colourless oil. This intermediate underwent azide reduction using Pd/C in EtOAc, forming crude 1-(tert-butyl)-2-methyl (2S,3R,4R)-4-amino-3-(3-(4,4,5,5-tetramethyl-1,3,2-dioxaborolan-2-yl)propyl)pyrrolidine-1,2-dicarboxylate (**66f**). Finally, **66f** was heated with 6 M HCl in a microwave reactor at 120 °C for 1 h to produce (2S,3R,4R)-4-amino-3-(3-boronopropyl)pyrrolidine-2-carboxylic acid (**66**) as a light brown solid. The same study described the synthesis of **67** (Scheme 29) using the intermediate **66c** (Scheme 28).

Caesium acetate and 18-crown-6 were added to a solution of intermediate **66c** in toluene stirred under N_2_ at r.t. This reaction yielded 1-(tert-butyl)-2-methyl (2S,3S,4R)-4-acetoxy-3-allylpyrrolidine-1,2-dicarboxylate (**67a**) as a colourless oil. In the next step, a mixture of pinacolborane, [Ir(cod)Cl]_2_, and dppe in DCM was prepared under N_2_ and stirred at r.t. The solution of **67a** was then added, producing 1-(tert-butyl)-2-methyl-(2S,3S,4R)-4-acetoxy-3-(3-(4,4,5,5-tetramethyl-1,3,2-dioxaborolan-2-yl)propyl)pyrrolidine-1,2-dicarboxylate (**67b**). For the final step, **67b** was deprotected by treatment with 12 M HCl in water, resulting in the formation of (2S,3S,4R)-3-(3-boronopropyl)-4-hydroxypyrrolidine-2-carboxylic acid (**67**) as a yellowish solid.

The synthesis of **68**, outlined in Scheme 30, began with the intermediate **68a**, prepared following established literature methods. Intermediate (2S,3S)-tert-butyl-3((benzyloxy)methyl)-1-(9-phenyl-9H-fluoren-9-yl)-3-(3-(4,4,5,5,-tetramethyl-1,3,2-dioxaborolan-2-yl)prpyl)azetidine-2-carboxylate (**68a**), used in the synthesis of various other compounds, was subjected to catalytic hydrogenation using 10% Pd/C in MeOH, yielding crude (2S,3S)-tert-butyl 3-(hydroxymethyl)-3-(3-(4,4,5,5-tetramethyl-1,3,3-di-oxaborolan-2-yl)propyl)azetidine-2-carboxylate (**68b**). This intermediate was used directly in the next step without further purification. To synthesize **68c**, TEA and BOC-anhydride were added to **68b** at 0 °C, resulting in (2S,3S)-di-tert-butyl 3-((benzyloxy)methyl)-3-(3-(4,4,5,5-tetramethyl-1,3,2-dioxaborolan-2-yl)propryl)azetidine-1,2-di-carboxylate (**68c**). Subsequent catalytic hydrogenation with 10% Pd/C in MeOH produced (2S,3S)-di-tert-butyl 3-(hydroxymethyl)-3-(3-(4,4,5,5-tetramethyl-1,3,2-dioxaborolan-2-yl)propryl)azetidine-1,2-dicarboxylate (**68d**), the corresponding hydroxymethyl derivative. Methanesulfonyl chloride and TEA were added to **68d** in DCM at 0 °C to form crude (2S,3S)-di-tert-butyl 3-(((methylsulfonyl)oxy)methyl)-3-(3-(4,4,5,5-tetramethyl-1,3,2-dioxaborolan-2-yl)propryl)azetidine-1,2-dicarboxylate (**68e**), a methylsulfonyl ester derivative. Without purification, **68e** was reacted with sodium azide in DMF at 80 °C for 15 h yielding (2S,3S)-di-tert-butyl 3-(azidomethyl)-3-(3-(4,4,5,5-tetramethyl-1,3,2-dioxaborolan-2-yl)propryl)azetidine-1,2-dicarboxylate (**68f**), an azide derivative. The oxidation of **68f** with NH_4_OAc and sodium periodate in a THF-H_2_O mixture at r.t. for 15 h yielded crude (3-((2S,3S)-3-(azidomethyl)-1,2-bis(tert-butoxycarbonyl)azetidine-3-l)prpyl)boronic acid (**68g**), a boronic acid derivative. This intermediate underwent reduction with PPh_3_ in THF—H_2_O at 60 °C under N_2_, forming (3-((2S,3S)-3-(aminomethyl)-1,2-bis(tert-butoxycarbonyl)azetidine-3-l)proyl)boronic acid (**68h**), an aminomethyl derivative. Finally, TFA was added to **68h** in DCM at 20 °C, deprotecting the BOC groups and yielding (2S,3S)-3-(aminomethyl)-3-(3-boronopropyl)azetidine-2-carboxylic acid (**68**) as the free base [67].

Mlynarski et al. reported the synthesis of many proline derivatives, including **69** (Scheme 31) and **70** (Scheme 32) [68].

The synthesis of **69** began with (2S, 4S)-1-tert-butyl-2-methyl-4-hydroxypyrrolidine-1,2-dicarboxylate (**69a**), which was treated with methanesulfonyl chloride and TEA in DCM at 0 °C. This yielded 1-(tert-butyl)-2-methyl(2S,4S)-4-((methylsulfonyl)oxy)pyrrolidine-1,2-dicarboxylate, an intermediate used directly in the next step. Reaction with sodium azide in DMF produced **69b** as a mixture of rotamers. A solution of NaOH in H_2_O was added to **69b** dissolved in THF/MeOH, generating (2S, 4R)-2-benzyl 1-tert-butyl-4-azidopyrrolidine-1,2-dicarboxylate (**69c**). This compound was treated with crotyl bromide in THF at –78 °C under N_2_, followed by KHMDS in toluene. The reaction mixture was warmed to r.t. and stirred for 3 h, yielding **69d** as a mixture of rotamers and E/Z olefins in a 78% yield. Next, **69d** was subjected to hydroboration using pinacolborane, [Ir(cod)Cl]_2_ and dppe in DCM under N_2_. Stirring overnight yielded **69e**, which was purified by SFC to separate the diastereoisomers. The major diastereoisomer **69f1** was identified as the anti-addition product, while the minor diastereomer **69f2** was the syn-addition product. (2R,4R)-2-benzyl 1-tert-butyl 4-azido-2-(4-(4,4,5,5-tetramethyk-1,3,2-dioxaborolan-2-yl)butyl)pyrrolidine-1,2-dicarboxylate (**69f2**) was subjected to catalytic hydrogenation using 10% Pd/C in a mixture of EtOAc and MeOH. This step produced **69g,** which was used without further purification. In the final step, **69g** was first treated with TFA in DCM at r.t. and then with 1 M HCl and phenylboronic acid in Et_2_O, obtaining (2R,4R)-4-amino-2-(4-boronobutyl)pyrrolidine-2-carboxilic acid (**69**) as a white solid.

Compound **70**, an analogue of **69** with a methyl amino group at position 4 of the proline ring, was synthesized starting from 1-(tert-butyl)2-methyl (2S,4R)-4-aminopyrrolidine-1,2-dicarboxylate (**70a**). The primary amino group in **70a** was protected using BOC-anhydride, and then methylated with NaH and CH_3_I in DMF to form intermediate **70c**. The synthesis from **70c** followed the same procedures as those for compound **69**, starting from the second step onward. The final product, (2R,4R)-2-(4-boronobutyl)-4-(methylamino)pyrrolidine-2-carboxylic acid (**70**), was obtained as a white solid.

## 5. Fourth-Generation Inhibitors

In this section of the review, we introduce a new class of compounds: the fourth-generation ARG inhibitors. These inhibitors are cyclic dipeptides composed of natural and non-natural amino acids, with the amino acids linked to a pyrrolidine or piperidine nitrogen, or an exocyclic amine group in proline residues [22]. The development of this new generation of ARG inhibitors was driven by the limitations of earlier compounds, which exhibited poor pharmacokinetic profiles, including very low oral bioavailability and a highly polar zwitterionic nature.

### 5.1. Peptide Boronic Acid Derivatives

#### 5.1.1. Peptide Cyclic Inhibitors

**Scheme 33:**
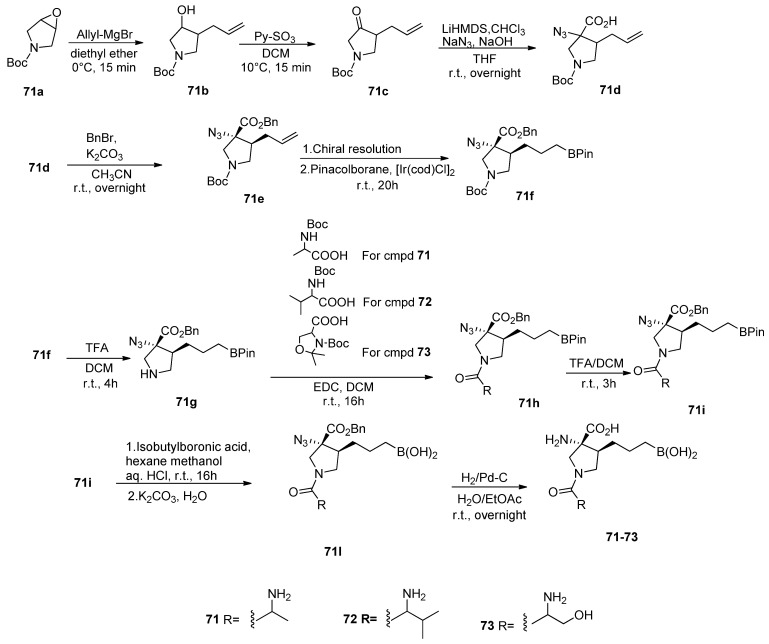
Synthesis of compounds **71**–**73**.

The fourth generation of inhibitors includes ABH derivatives, which feature a ring-constrained pyrrolidine that reduces entropy by positioning the quaternary amino acid in an optimal orientation for binding [48,54]. A notable example is numidargistat ((3R,4S)-3-amino-1-((S)-2-aminopropanoyl)-4-(3-boronopropyl)pyrrolidine-3-carboxylic acid (**71**), a potent ARG inhibitor with IC_50_ values of 86 nM for hARG-1 and 296 nM for hARG-2 [22]. In 2016, it was approved by the Food Drug Administration (FDA) for clinical trials to treat patients with metastatic solid tumours, both as a monotherapy and in combination with chemotherapy and immunotherapy [69]. Compound **71** and its analogues (**72**–**73**) were synthesized from Sjogren et al., with their synthetic protocols being patent-protected, as shown in Scheme 33 [70]. Specifically, compounds (3R,4S)-3-amino-1-((S)-2-amino-3-methylbutanoyl)-4-(3-boronopropylpyrrolidine)-3-carboxylic acid (**72**) and (3R,4S)-3-amino-1-((S)-2-amino-3-hydroxypropanoyl)-4-(3-boronopropyl)pyrrolidine-3-carboxylic acid (**73**) were prepared similarly to numidargistat (**71**), with the only difference being the reagents used with intermediate **71g**. For **72**, (tert-butoxycarbonyl)-L-valine was used, while for **73**, (S)-3-(tert-butoxycarbonyl)-2,2-dimethyloxazolidine-4-carboxylic acid was employed.

The synthesis started with tert-butyl 6-oxa-3-azabicyclo [3.1.0]hexane-3-carboxylate (**71a**), which was reacted with allyl-MgBr in a Grignard reaction to form compound **71b**. This was then oxidized using a sulphur trioxide pyridine complex (Py-SO_3_) to generate the ketone **71c**. Next, tert-butyl 3-allyl-4-oxopyrrolidine-1-carboxylate (**71c**) was treated with CHCl_3_ and LiHMDS, followed by NaN_3_ and NaOH to yield compound **71d**. The next step involved the protection of compound **71d** with BnBr and K_2_CO_3_, resulting in ((3R,4S)-3-benzyl 1-tert-butyl-4-allyl-3-azidopyrrolidine-1,3-dicarboxylate (**71e**), which was then subjected to hydroboration with pinacolborane and [Ir(cod)Cl]_2_ in DCM to produce compound **71f**. Compound **71f** underwent BOC deprotection with TFA in DCM, yielding **71g**. This was then alkylated with BOC-L-Alanine and EDC in DCM to form **71h**, which was deprotected again with TFA in DCM. In the final steps, compound **71i** was reacted with isobutylboronic acid, hexane, methanol, a solution of HCl, and K_2_CO_3_ to obtain the boronic derivative **71l.** Finally, hydrogenation with Pd/C resulted in the desired product, (3R,4S)-3-amino-1-((S)-2-aminopropanoyl)-4-(3-boronopropyl)pyrrolidine-3-carboxylic acid (**71**).

**Scheme 34:**
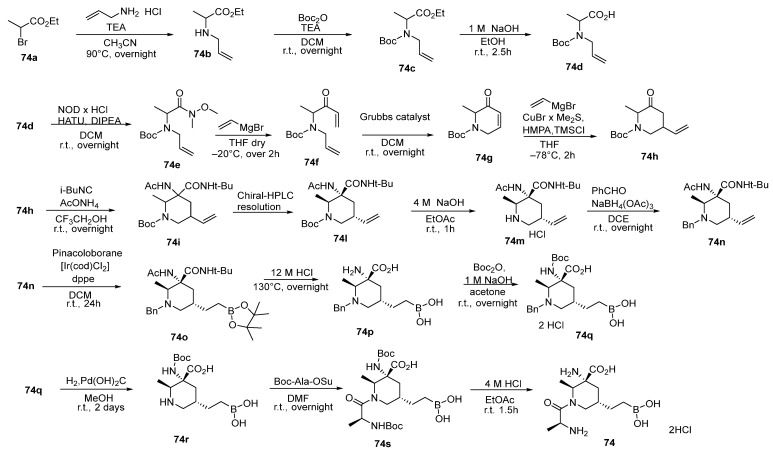
Synthesis of compound **74**.

An analogue of (2S,3R,5S)-3-amino-1-((S)-2-aminopropanoyl)-5-(2-boronoethyl)-2-methylpiperidine-3-carboxylic acid (**74**), which has a methyl group in position 2 of the piperidine ring, also exhibits activity against hARG-1 and hARG-2 with an IC_50_ of 249 nM or lower [22]. The full synthesis of this compound is detailed in Scheme 34 and is described briefly here.

The patented synthesis of compound **74** began with a substitution reaction between ethyl 2–bromopropionate (**74a**) and allylamine hydrochloride in the presence of TEA in ACN, yielding compound **74b**. This was followed by amine protection using BOC-anhydride, TEA, and DCM, stirred overnight at r.t., producing compound **74c**. Basic hydrolysis with NaOH and EtOH converted **74c** into N-allyl-N-(tert-butoxycarbonyl)alanine (**74d**). To a solution of **74d** in DCM, N,N-diisopropylethylamine (DIPEA), N,O–dimethylhydroxyamine, and HATU were added, forming the amide **74e** after overnight stirring at r.t. This intermediate underwent alkylation reaction with vinyl magnesium bromide in THF at –20 °C, followed by ring-closing metathesis using Grubbs catalyst 2nd generation in DCM, yielding cyclic compound **74g**. A second Grignard reaction with vinylmagnesium bromide and CuBr x Me_2_S in THF was performed, followed by the addition of tert-butyl-2-methyl -3-oxo-3,6-dihydropyridin–1(2H)–carboxylate (**74g**) and chlorotrimethyl silane at –78° C, which was then stirred overnight at r.t. to yield tert-butyl-2-methyl-3-oxo-5–vi nylpiperidine-1–carboxylate (**74h**). To a solution of **74h** in 2,2,2–trifluoroethanol, NH_4_OAc and tert- butylisocyanide were added dropwise, and the mixture was stirred at r. t. overnight. After chiral HPLC resolution, compound **74l** underwent a N-deprotection with HCl /AcOEt, yielding **74m**. This intermediate was protected with benzaldehyde, and sodium triacetoxyborohydride in DCE to obtain **74n** as a white foam. A hydroboration reaction was performed on **74n** using dppe, [Ir(cod)Cl]_2_, and pinacolborane in DCM, followed by treatment with 12 M HCl, resulting in the boronic acid derivative **74p**. This was protected using BOC anhydride, a solution 1M NaOH and acetone, producing compound **74q** as a white solid. The next step involved hydrogenation over 20% Pd (OH)_2_/C in MeOH, followed by N-alkylation with BOC-L-Ala-Osu in DMF, resulting in **74s**. In the final step, treatment of **74s** with a solution of 4 M HCl in EtOAc yielded the desired compound (2S,3R,5S)-3-amino-1-((S)-2-aminopropanoyl)-5-(2-boronoethyl)-2-methylpiperidine-3-carboxylic acid (**74**) [71].

**Scheme 35:**
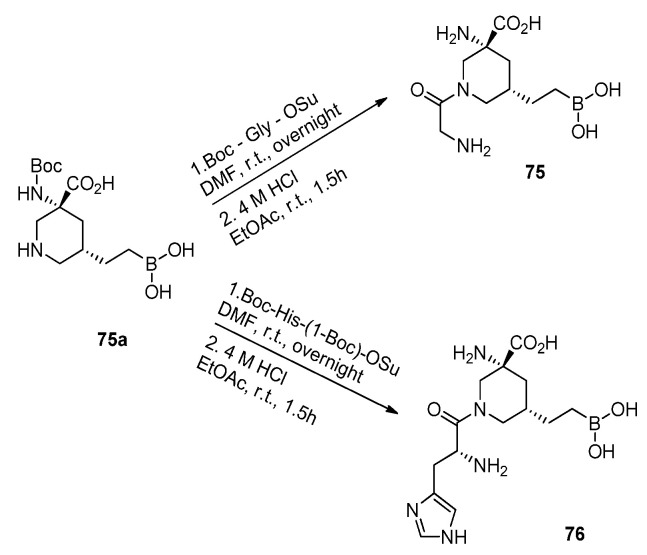
Synthesis of compounds **75**–**76**.

The compound (3R,5S)-3-amino-1-(2-aminoacetyl)-5-(2-boronoethyl)piperidine-3-carboxylic acid (**75**) is a glycine derivative with IC_50_ values ranging from 1 to 249 nM against hARG-1 and 500–999 nM against hARG-2. Its synthesis, outlined in Scheme 35, involves a patented two-step procedure. Starting from (3R, 5S)-5-(2-boronoethyl)-3-((tert-butoxycarbonyl)amino)piperidine-3-carboxylic acid (**75a**), Blaszczyk et al. performed an amidation reaction using BOC-Gly-OSu in DMF, followed by deprotection with 4 M HCI in EtOAC, yielding the final product **75** [22,71].

Another ARG inhibitor, (3R,5S)-1-(l-histidyl)-3-amino-5-(2-boronoethyl)piperidine-3-carboxylic acid trihydrochloride (**76**) is a histidine derivative with the same IC_50_ profile as **75** (1–249 nM for hARG-1 and 500–999 nM for hARG-2) [64]. Its synthesis follows the same procedure as **75** but uses BOC-L-His-(1-BOC)-Osu in the first step (Scheme 35).

**Scheme 36:**
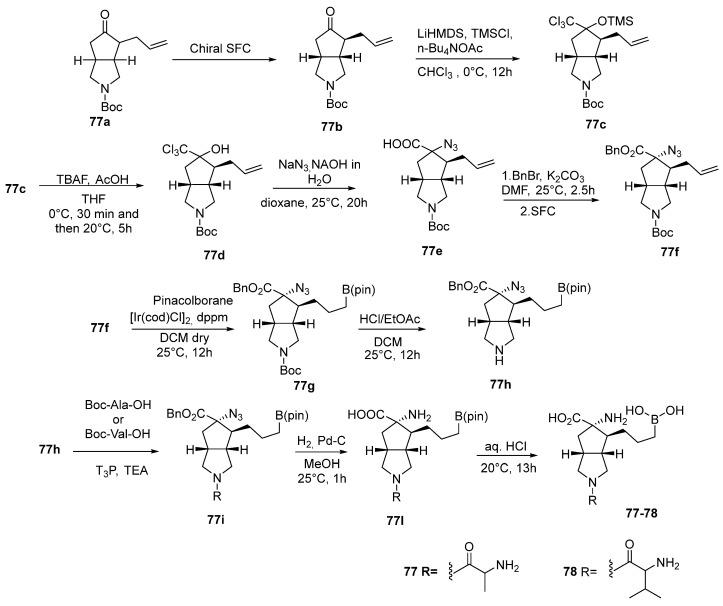
Synthesis of compounds **77**–**78**.

Among peptide inhibitors, notable examples include (3aR,4S,5S,6aR)-5-amino-2-((S)-2-aminopropanoyl)-4-(3 boronopropyl) octahydrocyclopenta[c] pyrrole-5-carboxylic acid (**77**) with an IC_50_ of 108 nM against hARG-1 (Merck,TOGA assay), and (3aR,4S,5S,6aR)-5-amino-2-((S)-2-amino-3-methylbutanoyl)-4-(3-boronopropyl)octahydrocyclopenta[c]pyrrole-5-carboxylic acid (**78**), containing a valine moiety and exhibiting an improved IC_50_ of 52 nM, (TOGA assay) [22]. The synthesis of both compounds follows a patented 10-step protocol outlined in Scheme 36 [62,63]. The processes are identical except for the coupling reagent used in the seventh step: BOC-Ala-OH for **77** and BOC-Val-OH for **78**.

The synthesis of compound **77** began with the resolution of **77a** via SFC, yielding tert-butyl-(3aR,4S,6aR)-4-allyl-5-oxohexahydrocyclopenta[c]pyrrolo-2(1H)-carboxylate (**77b**). In the second step, **77b** was treated with CHCl_3_, chlorotrimethylsilane, and LiHMDS (1 M in THF) under N_2_ at –78 °C, then warmed to –30 °C and stirred for 1.5 h. Following the addition of tetrabutylammonium acetate in DMF, the mixture was heated to 0 °C and stirred for 12 h, forming tert-butyl-(3aR,4S,6aR)-4-allyl-5-(trichloromethyl)-5-((trimethylsilyl)oxy)hexahydrocyclopenta[c]pyrrole-2(1H)-carboxylate (**77c**). In the third step, the product was treated with acetic acid and tetra-n-butylammonium fluoride in THF, to produce **77d**, which was then reacted with sodium azide and sodium hydroxide in water to give (3aR,4S,6aR)-4-allyl-5-azido-2-(tert-butoxycarbonyl)octahydrocyclopenta[c]pyrrole-5-carboxylic acid (**77e**). The benzylation of **77e** with K_2_CO_3_ and benzyl bromide yielded 5-benzyl 2-(tert-butyl)(3aR,4S,5S,6aR)-4-allyl-5-azidohexahydrocyclopenta[c]pyrrole-2,5(1H)-dicarboxylate (**77f**). A hydroboration reaction with [Ir(cod)Cl]_2_ and pinacolborane in DMC then produced 5-benzyl 2-(tert-butyl) (3aR,4S,5S,6aR)-5-azido-4-(3-(4,4,5,5-tetramethyl-1,3,2-dioxaborolan-2-yl)propyl)hexahydro cyclopenta[c]pyrrole-2,5(1H)-dicarboxylate (**77g**), which was deprotected with 4 M HCl in EtOAc, yielding benzyl(3aR,4S,5S,6aR)-5-azido-4-(3-(4,4,5,5-tetramethyl-1,3,2-dioxaborolan2-yl)propyl)octahydrocyclopenta[c]pyrrole-5-carboxylate (**77h**). Compound **77h** was N-alkylated with BOC-Ala-OH or BOC-Val-OH in DMF and in the presence of propanephosphonic acid anydride (T_3_P) and TEA to form (3aR,4S,5S,6aR)-5-azido-2-((tert-butoxycarbonyl)-L-alanyl)-4-(3-(4,4,5,5-tetramethyl-1,3,2-dioxaborolan-2-yl)propyl)octahydrocyclopenta[c]pyrrole-5-carboxylate or (3aR,4S,5S,6aR)-5-azido-2-((tert-butoxycarbonyl)-L-valin)-4-(3-(4,4,5,5-tetramethyl-1,3,2-dioxaborolan-2-yl)propyl)octahydrocyclopenta[c]pyrrole-5-carboxylate (**77i**). Subsequent deprotection with H_2_ over Pd-C gave **77l**, which was used directly in the final step. The treatment of **77l** with 6 M HCl at 20 °C for 13 h yielded the final product (3αR,4S,5S,6αR)-2-(L-alanyl)-5-amino-4-(3-boronopropyl)octahydrocyclopenta[c]pyrrole-5-carboxylic acid (**77**) and (3αR,4S,5S,6αR)-2-(L-vanil)-5-amino-4-(3-boronopropyl)octahydrocyclopenta[c]pyrrole-5-carboxylic acid (**78**) as white solids (TFA salt).

#### 5.1.2. Proline Cyclic Inhibitors

**Figure 5:**
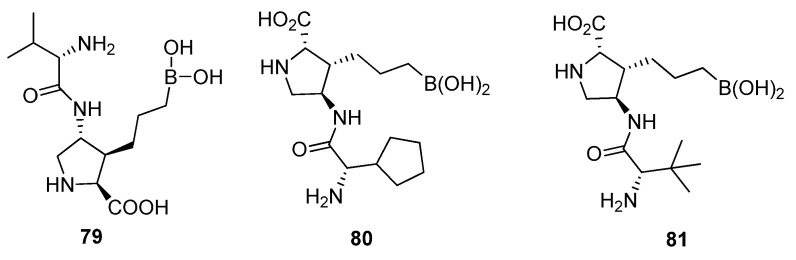
Chemical structures of compounds **79**–**81**.

Other peptide inhibitors of ARG are based on a proline scaffold, where the amino group of the proline core is coupled with various natural amino acids such as valine, leucine, and proline, or unnatural amino acids like cyclopentylglycine, tert-leucine, and indanylglycine. Examples include (2S,3R,4R)-4-((S)-2-amino-3-methylbutanamido)-3-(3-boronopropyl)pyrrolidine-2-carboxylic acid (**79**), (2S,3R,4R)-4-((S)-2-amino-2-cyclopentylacetamido)-3-(3-boronopropyl)pyrrolidine-2-carboxylic acid (**80**), and (2S,3R,4R)-4-((S)-2-amino-3,3-dimethylbutanamido)-3-(3-boronopropyl)pyrrolidine-2-carboxylic acid (**81**). These compounds, reported in Figure 5, exhibit improved inhibitory properties.

**Scheme 37:**
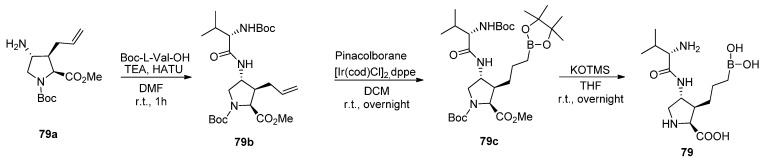
Synthesis of compound **79**.

Compound **79**, featuring an isopropyl group as a substituent, inhibits hARG-1 with an IC_50_ = 320 nM and hARG-2 with IC_50_ = 330 nM (TOGA assay). In contrast, compounds **80** and **81**, containing cyclopentylglycine and tert-leucine as unnatural amino acids linked to the proline scaffold, are significantly more potent, inhibiting hARG-1 with an IC_50_ = 0.8 nM (TOGA assay) [22]. Compounds **80** and **81** were synthesized following the same method as compound **79** (Scheme 37) but using appropriate starting materials [72].

The patented synthesis of compound **79**, described by Achab AA et al., involves three steps [72]. Starting with (2S,3R,4R)-1-tert-butyl 2-methyl 3-allyl-4-aminopyrrolidine-1,2-dicarboxylate (**79a**), the compound undergoes N-alkylation using BOC-L-Val-OH, TEA, and HATU, yielding intermediate **79b**. This is followed by a hydroboration reaction with pinacolborane, [Ir(cod)Cl]_2_, and dppe to produce compound **79c**. In the final step, the intermediate is treated with potassium trimethylsilanolate, then with 6 M HCl, to obtain the target compound (2S,3R,4R)-4-((S)-2-amino-3-methylbutanamido)-3-(3-boronopropyl)pyrrolidine-2-carboxylic acid (**79**).

**Scheme 38:**
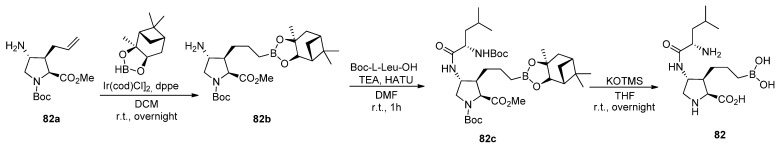
Synthesis of compound **82**.

Another ARG inhibitor is (2S,3R,4R)-4-((S)-2-amino-4-methylpentanamido)-3-(3-boronopropyl)pyrrolidine-2-carboxylic acid (**82**), which incorporates leucine and the natural amino acid attached to the amine group of the proline scaffold. This compound inhibits hARG-1 with an IC_50_ = 1.6 nM (TOGA) and is synthesized in three steps, as outlined in Scheme 38 [22,72]. Starting with 1-(tert-butyl)-2-methyl(2S,3R,4R)-3-allyl-4-aminopyrrolidine-1,2-dicarboxylate (**82a**), a hydroboration reaction with a pinanediolborane derivative, (3aR,4R,6R,7aS)-3a,5,5-trimethylhexahydro-4,6-methanobenzo[d][1,3,2]dioxaborole, [Ir(cod)Cl]_2_, and dppe yields compound **82b**. This intermediate is then N-alkylated using BOC-L-Leu-OH, TEA, and HATU to produce compound **82c**. In the final step, compound **82c** reacts with potassium trimethylsilanolate, followed by treatment with 6 M HCl, resulting in the desired compound, (2S,3R,4R)-4-((S)-2-amino-4-methylpentanamido)-3-(3-boronopropyl)pyrrolidine-2-carboxylic acid (**82**).

**Scheme 39:**
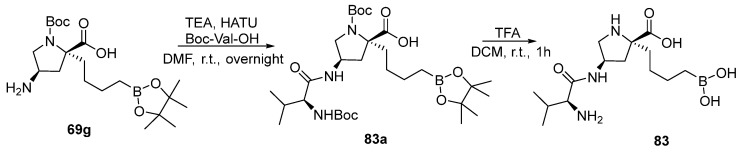
Synthesis of compound **83**.

The compound (2R,4R)-4-((S)-2-amino-3-methylbutanamido)-2-(4-boronobutyl)pyrrolidine-2-carboxylic acid (**AZD0011**, **83**), a valine analogue, exhibited IC_50_ values of 320 nM against hARG-1 and 330 nM against hARG-2, as determined by the TOGA assay. Furthermore, pharmacological tests using the mouse xenograft model revealed that **83** acted as prodrug, releasing the parent compound **69** in vivo [73]. The synthesis of **83**, outlined in Scheme 39 [62], involved two steps starting from (2R,4R)-4-amino-1-(tert-butoxycarbonyl)-2-(4-(4,4,5,5-tetramethyl-1,3,2-dioxaborolan-2-yl)butyl)pyrrolidine-2-carboxylic acid (**69g**). In the first step, **69g** underwent N-alkylation with BOC-Val-OH, TEA, and HATU yielding intermediate **83a**. The second step involved deprotection using TFA, Et_2_O, a solution 1 M HCl, and phenylboronic acid, producing the final compound (2R,4R)-4-((S)-2-amino-3-methylbutanamido)-2-(4-boronobutyl)pyrrolidine-2-carboxylic acid (**83**).

**Scheme 40:**
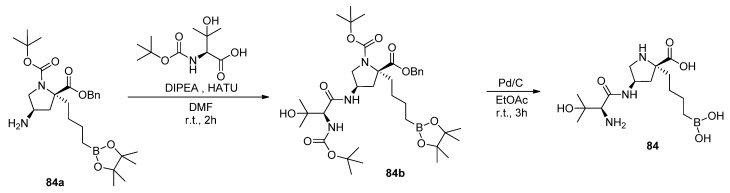
Synthesis of compound **84**.

The compound (2R,4R)-4-((S)-2-amino-3-hydroxy-3-methylbutanamido)-2-(4-boronobutyl)pyrrolidine-2-carboxylic acid (**84**), a hydroxyvaline analogue, displayed IC_50_ values of 340 nM and 520 nM against hARG-1 and hARG-2, respectively, as determined by the TOGA assay [22]. Its synthesys outlined in Scheme 40, involved two steps starting with (2R,4R)-2-benzyl-1-tert-butyl 4-amino-2-(4-(4,4,5,5-tetramethyl-1,3,2-dioxaborolan-2-yl)butyl)pyrrolidine-1,2-dicarboxylate (**84a**). The first step was N-alkylation with (S)-N-α-tert-butoxycarbonyl-3,3-dimethyl-serine, DIPEA, and HATU, yielding intermediate **84b**. In the second step, deprotection was achieved using Pd/C in EtOAc, followed by treatment with TFA, Et_2_O, a solution 1 M HCl, and phenylboronic acid, producing the final compound (2R,4R)-4-((S)-2-amino-3-hydroxy-3-methylbutanamido)-2-(4-boronobutyl) pyrrolidine-2-carboxylic acid (**84**) [68].

As observed in the structures of the fourth-generation ARG inhibitors presented thus far, the nitrogen atom is linked to an amino acid of a varying nature through a peptide bond. In some cases, the design of these compounds has resulted in improvements in both activity and pharmacokinetic profiles, fulfilling the objectives for which this generation was developed. However, in other instances, these inhibitors exhibited lower inhibitory activity compared to the non-peptide derivatives of the third generation and could only be considered prodrugs in specific cases. For example, it has been demonstrated that peptide derivatives, such as compound **83**, were rapidly metabolized in vivo through the hydrolysis of the peptide bond, releasing the non-peptide derivative **69**, which exhibited superior activity.

**Scheme 41:**
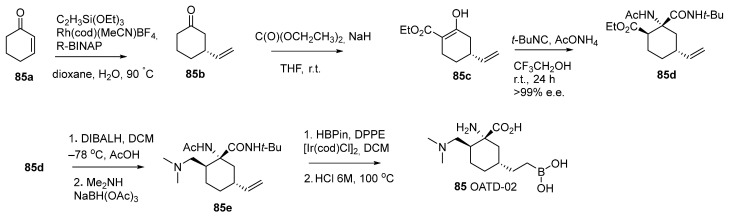
Synthesis of compound **85**.

**OATD-02** (**85**), developed by OncoArendi Therapeutics (now Molecure), is a boronic acid derivative and an intracellular dual inhibitor of ARG-1 and ARG-2, with potent IC_50_ values of 20 nM and 48 nM for hARG-1 and -2, respectively [22]. Currently in preclinical development [74], its synthesis involves five steps, as detailed in Scheme 41 [28,75]. Starting from 2-cyclohexen-1-one (**85a**), the reaction with triethoxyvinylsilane under the catalysis of Rh(cod)(MeCN)_2_BF_4_ and R-BINAP in dioxane produced intermediate **85b**. Further processing with sodium hydride and diethyl carbonate yielded **85c**, which was converted to **85d** by reaction with NH_4_OAc in 2,2,2-trifluoroethanol and tert-butyl isocyanide. The subsequent reduction of (1R,2R,4R)-2-acetamido-2-(tert-butylcarbamoyl)-4-vinylcyclohexane-1-carboxylate (**85d**) with a solution 1 M DIBAL-H in DCM at –78 °C, followed by the addition of dimethylamine in THF and sodium triacetoxyborohydride, led to the formation of (1R,2S,5R)-1-acetamido-N-(tert-butyl)-2-((dimethylamino)methyl)-5-vinylcyclohexanecarboxamide (**85e**). Reacting **85e** with pinacolborane in the presence of dppe and [Ir(cod)Cl]_2_, followed by reflux in 6 M HCl, yielded the crude product. Purification through chromatography on the silica gel C-18 (isocratic elution, H_2_O), and, next, flash chromatography on the DOWEX^®^ 50WX8 ion exchange resin (eluent 0.1 M ammonia in water) produced the final compound (OATD-02) **85**.

## 6. Side Chain β-Substituted ABH Analogues

**Figure 6:**
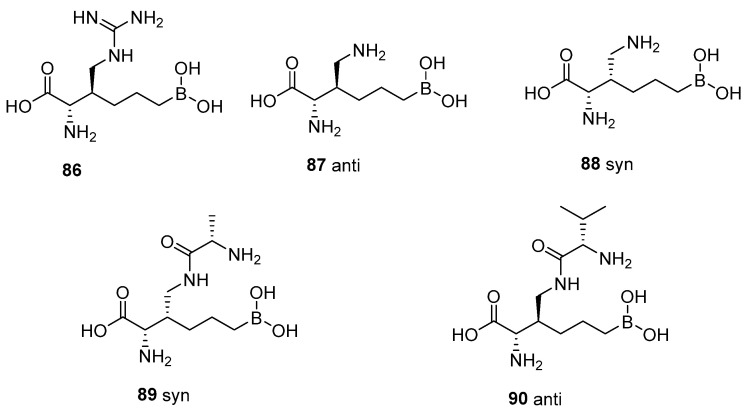
Chemical structures of compounds **86**–**90**.

Recently, Shields and colleagues developed novel ARG inhibitors by exploring the previously little-studied β-position of the compound ABH (**9**) [76]. Specifically, they synthesized promising inhibitors by alkylating the β-position of ABH with various functionalized alkyl chains to increase potency. Until now, companies and research groups had focused primarly on substitution at the α-position or the simultaneous substitution of both the α- and β-positions through cyclization. Through their chemical modifications, Shields et al. have extensively occupied the channel leading to the active site by targeting the β-position, without relying on cyclization. Among the twenty compounds synthesized, the most potent inhibitors able to inhibit ARG-1 with an IC_50_ value lower than that ABH (i.e., lower than the 470 nM obtained from the biochemical assays performed by the same authors) were compounds **86–88**, shown in Figure 6. Notably, compounds **87** and **88** shared the same chemical structure but differed in spatial arrangement: 87 was the anti derivative, and 88 was the syn derivative. The anti arrangement (**87**) exhibited higher potency. The X-ray crystallography of the **87** and 88-ARG-2 complexes prompted the authors to add an amino acid residue to **87** and **88**, forming a peptide bond to further improve potency. Various amino acid residues were tested, and the most promising inhibitors were derivatives **89** and **90** (Figure 6). For the syn compound, alanine was added while valine was added to the anti compound. Interestingly, the syn compound (**88**) became more potent after alanine was added (**89**), surpassing both **87** and its corresponding derivative, 90. Moreover, pharmacological tests confirmed that **89** and **90** acted as prodrugs, releasing the respective parent compounds in vivo [76].

**Scheme 42:**
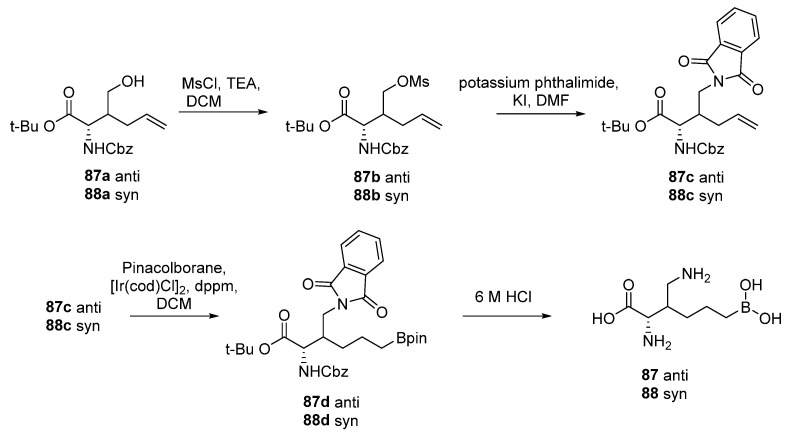
Synthesis of compounds **87–88**.

The synthetic procedure of compounds **87** and **88** is shown in Scheme 42.

The synthesis began with the mesylation of *tert*-butyl (2S,3S)-2-(((benzyloxy)carbonyl)amino)-3-(hydroxymethyl)hex-5-enoate (**87a**) and *tert*-butyl (2S,3R)-2-(((benzyloxy)carbonyl)amino)-3-(hydroxymethyl)hex-5-enoate (**88a**) and the subsequent protection of the amino function using potassium phthalimide to give the corresponding protected amines *tert*-butyl (2S,3R)-2-(((benzyloxy)carbonyl)amino)-3-((1,3-dioxoisoindolin-2-yl)methyl)hex-5-enoate (**87c**) and *tert*-butyl (2S,3S)-2-(((benzyloxy)carbonyl)amino)-3-((1,3-dioxoisoindolin-2-yl)methyl)hex-5-enoate (**88c**). Subsequently, the hydroboration reaction of **87c** and **88c** was performed in the presence of [Ir(cod)Cl]_2_, dppm and pinacolborane yielding **87d** and **88d**, respectively. In the last step, a global deprotection in acidic medium was performed to remove the benzyl carbamate, *tert*-butyl ester, and the pinacol-protecting groups, revealing the final compounds (2S,3R)-2-amino-3-(aminomethyl)-6-boronohexanoic acid dihydrochloride (**87**) and (2S,3S)-2-amino-3-(aminomethyl)-6-boronohexanoic acid dihydrochloride (**88**) as hydrochloride salts. These salts were freebased by passage through an ion exchange column and then freeze-dried.

## 7. Natural Compounds as ARG Inhibitors

Plant-derived compounds, often inspired by traditional medicine, offer a promising avenue for discovery ARG inhibitors. Natural and semi-synthetic compounds not only enhance molecular diversity but also hold the potential to reduce toxicity [34,77].

Among natural ARG inhibitors, polyphenols stand out for their bioactivity and safety. This class includes flavonoids, phenolic acids, and tannins, which have demonstrated significant ARG inhibitory activity and could serve as valuable lead compounds. On the semi-synthetic side, cinnamide derivatives, derived from cinnamic acid, show great potential. These compounds can be chemically modified to improve their potency and selectivity. By combining natural and semi-synthetic approaches, researchers can create a robust platform for developing new ARG inhibitors, benefiting from molecular diversity, reduced toxicity, and structure–activity relationship (SAR) insights.

### 7.1. Polyphenols

Polyphenols are among the most prominent natural compounds identified as ARG inhibitors. These secondary plant metabolites, abundant in fruits, vegetables, and medicinal plants, offer a wide array of health benefits. Key classes of polyphenols include flavonoids, phenolic acids, stilbenes, and lignans, many of which have demonstrated ARG-inhibitory activity, partly linked to their influence on NO production.

Notable examples (Table 2) include chlorogenic acid (**91**) (a phenolic acid), picetannol (**92**) and resveratrol (**93**) (stilbens), and taxifolin (**94**) (flavanoid). These compounds showed IC_50_ values of 10.6 μM, 12.1 μM, 18.2 μM, and 23.2 μM, respectively, against ARG-1 in assays using mammalian bovine liver [78]. Piceatannol-3′-O-β-D-glucopyranoside (**95**), a stilbene glycoside, inhibited both ARG-1 and ARG-2 in a dose-dependent manner with IC_50_ values of 11.2 μM and 11.0 μM, measured in rat liver and kidney lysates, respectively [79]. Studies by Arraki et al. [80] highlighted several polyphenols, including ellagic acid (**96**),various luteolin derivatives extracted from the leaves of Morus alba, like luteolin-7-diglucoside (**97**), luteolin-7-glucoside (**98**), and luteolin (**99**), as well as stilbenes like scirpusin B (**100**), ε-viniferin (**101**), cyperusphenol B (**102**), carexinol A (**103**), and the newly identified virgatanol (**104**) stilbenes and polyphenols isolated from various species of Cyperus and Carex, from the Cyperaceae family—which showed inhibitory effects on purified bovine liver (Table 2). Additionally, Sauchinone (**105**), isolated from Saurus chinensis extract, showed an IC_50_ of 61.4 μM against ARG-2 in murine kidney lysates [81]. Methanolic extracts of Scutellaria indica also showed promise, with compounds (2S)-5,7-dihydroxy-8,20-dimethoxyflavanone (**106**) and (2S)-5,20,50-trihydroxy-7, 8-dimethoxyflavanone (**107**) displaying IC_50_ values of 25.1 μM and 11.6 μM, respectively, against ARG-2 [82]. However, comparing these findings is challenging due to the variability in assay conditions. Despite this limitation, structural analyses suggest that features like the caffeoyl (3,4-dioxycinnamoyl) group and catechol functionality are crucial for ARG inhibition. Although the identified natural compounds mostly exhibit micromolar-range activity, these insights provide a foundation for designing more potent ARG inhibitors and expanding the chemical space of natural product-derived therapeutics.

### 7.2. Cinnamide Derivatives

Pham et al. simplified the structure of chlorogenic acid—an ester of caffeic and quinic acid—to create compound (**108**) (Table 2), also known as caffeic acid phenylamide (CAPA). In tests using a micro-assay on purified bovine liver arginase (bARG-1), CAPA showed slightly better activity than chlorogenic acid, with IC_50_ values of 6.9 µM and 10.6 µM, respectively. However, when tested on recombinant human arginase (hARG-1), CAPA’s activity decreased significantly, with an IC_50_ of 60.3 µM.

This study highlights the value of using bARG-1, a cost-effective alternative, for initial screening of potential mammalian ARG inhibitors. However, it also emphasizes the importance of testing promising compounds on hARG-1 to ensure their relevance before advancing to further studies. The research identified the cinnamoyl group and catechol moiety as essential structural features for inhibitory activity. Despite CAPA’s relatively high IC_50_ against hARG-1, its structure points to cinnamide derivatives as promising lead compounds for developing therapeutically effective ARG inhibitors [83].

## 8. Conclusions and Future Perspectives

In conclusion, ARG, the enzyme responsible for the metabolism of the amino acid L-arginine, plays a key role in numerous pathophysiological processes, making it a significant target for researchers aiming to combat various disorders. Although the enzyme was discovered many years ago, recent advancements have spurred renewed research into ARG and its inhibitors, leading to the development of new molecules designed to target this enzyme. This review traces the evolution of ARG inhibitors, categorizing them into different generations and classes based on structure variations, including recently developed molecules and those derived from natural or semi-synthetic sources. For the first time, in this review the structures and syntheses of ARG inhibitors described in patent applications have been reported.

As emphasized throughout the review, various pathologies are characterized by the uncontrolled expression of one of the two ARG isoforms, underscoring the need for selective inhibitors targeting either ARG-1 or ARG-2. Despite numerous studies and clinical trials demonstrating the potential of ARG as a biomarker and diagnostic tool for cancer progression, there is still a need for standardized clinical definitions of ARG activity and for the consistent measurement of ARG-1 and ARG-2 expression levels in blood or tissues for cancer diagnosis. To address this challenge, an artificial intelligence-based prediction model could be developed, leveraging deep learning of clinical data, including cancer types, ARG activity values, expression levels, and patient information, to assess cancer progression.

Despite significant progress, a fully selective inhibitor remains elusive due to the structural similarities between the two isoforms. Notable examples include numidargistat and OATD-02, although neither shows significant selectivity for one isoform over the other. However, OATD-02 offers a unique advantage as the first potent dual ARG-1/ARG-2 inhibitor in its class. Its enhanced antitumor activity in vivo has been attributed to its complex mechanism of action, targeting both intracellular and extracellular ARGs. Consequently, OATD-02 stands out as the only pharmacological tool capable of effectively harnessing the benefits of inhibiting both isoenzymes, while also regulating also CD8^+^ and Treg cell activity in contrast to numidargistat.

Both numidargistat and OATD-02 have advanced to the clinical phase, ref. [84] with OATD-02 still in the recruitment phase. Numidargistat has been evaluated in five separate phase I studies for advanced or metastatic solid tumours, primarily in combination with other immunotherapies or conventional chemotherapies. However, the literature suggests that immunochemotherapy has shown superior efficacy compared to immunotherapy alone [85]. While numidargistat has a manageable safety profile, characterized primarily by inhibition of the urea cycle, immune-related adverse events, and a pharmacodynamic increase in plasma arginine levels [86,87], it has not yet advanced beyond phase I due to a response below expectations in terms of overall response rate and disease control rate. Another ARG inhibitor in clinical trials, CB-280 (structure undisclosed), is being tested for cystic fibrosis [88]. This compound has shown good tolerability, with no dose-limiting toxicities or severe grade adverse reactions [89]. In addition to small molecule inhibitors, a peptide vaccine targeting ARG-1 has been tested in clinical studies demonstrating a good safety profile, with no severe adverse reactions and with 90% of patients showing a measurable immune response to the peptide, though the clinical antitumor response has been modest [90]. Further details on the clinical studies have been published in Failla et al. [31].

The challenge of developing selective inhibitors for the ARG isoforms remains highly relevant. Structural modifications, such as incorporating boronic acid in place of the guanidine group and side chain derivatizations, have led to significant improvements, though an ideal compound has yet to be achieved. High-throughput screening offers a promising solution, especially when the target structure is not fully defined, and it can be used alongside computational strategies.

Moreover, improving the pharmacokinetic profile of these molecules presents another challenge. A promising approach involves leveraging medicinal chemistry techniques to achieve the desired selectivity for one of the two isoforms while simultaneously enhancing pharmacokinetic properties, as discussed in our previous perspective [31]. Strategies such as the prodrug approach, utilizing small molecules or polymers as carriers, could improve drug targeting [91]. Alternatively, molecular hybridization techniques hold potential for improving pharmacokinetic profile of ARG inhibitors, thereby boosting their pharmacological activity [92,93,94]. Furthermore, since ARG inhibitors have shown promise as probes for molecular imaging, improving their selectivity and biopharmaceutical properties could pave the way for both therapeutic and diagnostic applications.

## Figures and Tables

**Table 1:** Chemical structures of compounds **21**–**46**.

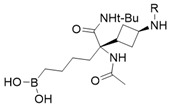		
Compound	R	IC_50_
**21 ** (S)-2-amino-6-borono-2-(1S,3R)-3-(3-phenylpropylamino)cyclobutyl)hexanoic acid	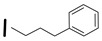	0.1–25 nM (hARG-1) 26–100 nM (hARG-2)
**22** (S)-2-amino-6-borono-2-((1S,3R)-3-(3-(3-chloro-5fluorophenyl)propylamino) cyclobutyl)hexanoic acid	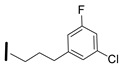	26–100 nM (hARG-1) 0.1–25 nM (hARG-2)
**23 ** (S)-2-amino-6-borono-2-((1s,3R)-3-(3-(3,4-difluorophenyl)propylamino)cyclobutyl) hexanoic acid	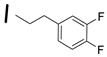	26–100 nM (hARG-1) 26–100 nM (hARG-2)
**24** (S)-2-amino-6-borono-2-((1S,3R)-3-(3-(2,4-dichlorophenyl)propylamino)cyclobutyl)hexanoic acid	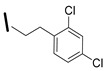	26–100 nM (hARG-1) 26–100 nM (hARG-2)
**25 ** (S)-2-amino-6-borono-2-((1S,3R)-3-(2,3-dihydro-1h-inden-2yl-amino)cyclobutyl)hexanoic acid	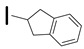	0.1–25 nM (hARG-1) 26–100 nM (hARG-2)
**26 ** (S)-2-amino-6-borono-2-((1S,3R)-3-(4-*tert*-butylbenzylamino)cyclobutyl)hexanoic acid	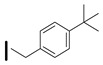	26–100 nM (hARG-1) 26–100 nM (hARG-2)
**27 ** (S)-2-amino-2-((1S,3R)-3-(biphenyl-3-ylmethylamino)cyclobutyl)-6-boronohexanoic acid	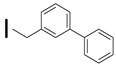	0.1–25 nM (hARG-1) 26–100 nM (hARG-2)
**28** (S)-2-amino-6-borono-2-((1S,3R)-3-((4′-(trifluoromethyl)biphenyl-3-yl) methylamino)cyclobutyl)hexanoic acid	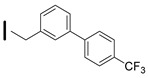	26–100 nM (hARG-1) 26–100 nM (hARG-2)
**29 ** (S)-2-amino-6-borono-2-((1S,3R)-3-((4′-chlorobiphenyl-3yl)methylamino)cyclobutyl)hexanoic acid	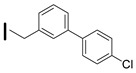	0.1–25 nM (hARG-1) 0.1–25 nM (hARG-2)
**30 ** (S)-2-amino-6-borono-2-((1S,3R)-3-((4-fluoronaphthalen-1yl)methylamino)cyclobutyl)hexanoic acid	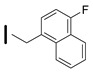	0.1–25 nM (hARG-1) 0.1–25 nM (hARG-2)
**31 ** (S)-2-amino-6-borono-2-((1S,3R)-3-((5-fluoronaphthalen-1yl)methylaminocyclobutyl)hexanoic acid	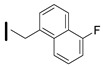	0.1–25 nM (hARG-1) 26–100 nM (hARG-2)
**32** (S)-2-amino-2-(1S,3R)-3-(anthracen-9-ylmethylamino)cyclobutyl)-6-borono hexanoic acid	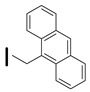	26–100 nM (hARG-1) 0.1–25 nM (hARG-2)
**33 ** (S)-2-amino-6-borono-2-((1S,3R)-3-(2-morpholinobenzylamino)cyclobutyl)hexanoic acid	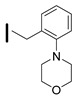	26–100 nM (hARG-1) 26–100 nM (hARG-2)
**34** (S)-2-amino-6-borono-2-((1R,3R)-3-(((S)-1,2,3,4-tetrahydroisoquinolin-3-yl)methylamino)cyclobutyl)hexanoic acid	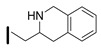	26–100 nM (hARG-1) 26–100 nM (hARG-2)
**35** (S)-2-amino-6-borono-2-((1S,3R)-3-((2,3-dihydrobenzofuran-5-yl)methylamino)cyclobutyl)hexanoic acid	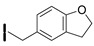	26–100 nM (hARG-1) 26–100 nM (hARG-2)
**36 ** (S)-2-amino-6-borono-2-((1S,3R)-3-((3′,4′-dichlorobiphenyl-4yl)methylamino)cyclobutyl)hexanoic acid	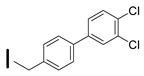	0.1–25 nM (hARG-1) 0.1–25 nM (hARG-2)
**37** (S)-2-amino-6-borono-2-((1S,3R)-3-((4′-chlorobiphenyl-4yl)methylamino)cyclobutyl)hexanoic acid	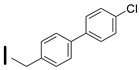	0.1–25 nM (hARG-1) 0.1–25 nM (hARG-2)
**38** (S)-2-amino-6-borono-2-((1S,3R)-3-((4′-(trifluoromethyl)biphenyl-4-yl)methylamino)cyclobutyl) hexanoic acid	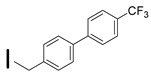	0.1–25 nM (hARG-1) 0.1–25 nM (hARG-2)
**39 ** (S)-2-amino-6-borono-2-((1S,3R)-3-((4′-fluorobiphenyl-4yl)methylamino)cyclobutyl)hecanoic acid	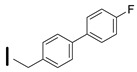	0.1–25 nM (hARG-1) 26–100 nM (hARG-2)
**40 ** (S)-2-amino-6-borono-2-((1S,3R)-3-(4-hydroxybenzylamino)cyclobutyl)hexanoic acid	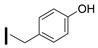	26–100 nM (hARG-1) 26–100 nM (hARG-2)
**41** (S)-2-amino-6-borono-2-((1S,3R)-3-(4-(4-chlorophenoxy)benzylamino)cyclobutyl)hexanoic acid	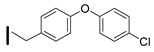	0.1–25 nM (hARG-1) 0.1–25 nM (hARG-2)
**42** (S)-2-amino-6-borono-2-(1S,3R)-3-((4′-chlorobiphenyl-2-yl)methylamino)cyclobutyl)hexanoic acid	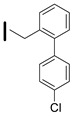	26–100 nM (hARG-1) 26–100 nM (hARG-2)
**43** (S)-2-amino-6-borono-2-((1S,3R)-3-((6-phenylpyridin-3-yl)methylamino)cyclobutyl)hexanoic acid	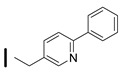	26–100 nM (hARG-1) 0.1–25 nM (hARG-2)
**44 ** (S)-2-((1S,3R)-3-((9h-fluoren-2-yl)methylamino)cyclobutyl)-2-amino-6-borono-hexanoic acid	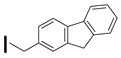	0.1–25 nM (hARG-1) 0.1–25 nM (hARG-2)
**45** (S)-2-amino-6-borono-2-((1S,3R)-3-((4′-(trifluoromethyl)biphenyl-2 yl)methylamino)cyclobutyl)hexanoic acid	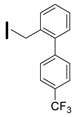	26–100 nM (hARG-1) 26–100 nM (hARG-2)
**46 ** (S)-2-amino-6-borono-2-((1S,3R)-3-(4-cyclohexylbenzylamino)cyclobutyl)hexanoic acid	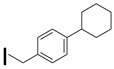	0.1–25 nM (hARG-1) 26–100 nM (hARG-2)

**Table 2:** Structures and IC_50_ values of natural and semi-synthetic compounds.

Compound	Structure	ARG-1 Activity	ARG-2 Activity
**91** Chlorogenic acid	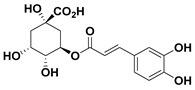	IC_50_ = 10.6 µM ^a^	
**92** Picetannol	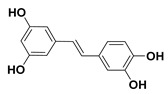	IC_50_ = 12.1 µM ^a^	
**93** Resveratrol	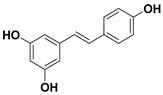	IC_50_ = 18.2 µM ^a^	
**94** Taxifolin	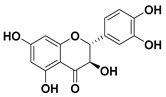	IC_50_ = 23.2 µM ^a^	
**95** Piceatannol-3′-O-β-D-glucopyranoside	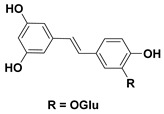	IC_50_ = 11.22 µM ^b^	IC_50_ = 11.06 µM ^c^
**96** Ellagic acid	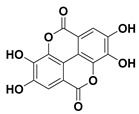	IC_50_ = 78.9 µM ^a^	
**97** Luteolin-7-diglucoside	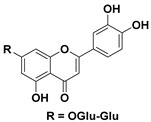	IC_50_ = 152.4 µM ^a^	
**98** Luteolin-7-glucoside	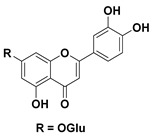	IC_50_ = 99.4 µM ^a^	
**99** Luteolin	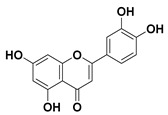	IC_50_ = 95.3 µM ^a^	
**100** Scirpusin B	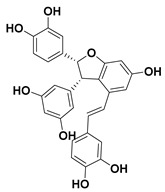	IC_50_ = 22.6 µM ^a^	
**101** ε-Viniferin	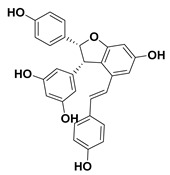	IC_50_ = 27.8 µM ^a^	
**102** Cyperusphenol B	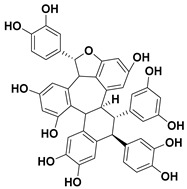	IC_50_ = 12.2 µM ^a^	
**103** Carexinol A	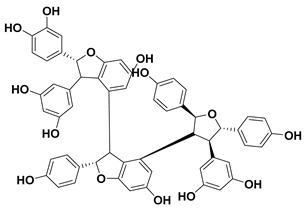	IC_50_ = 25.3 µM ^a^	
**104** Virgatanol	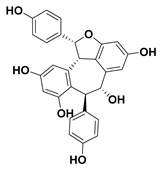	IC_50_ = 182.1 µM ^a^	
**105** Sauchinone	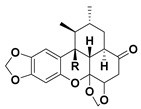		IC_50_ = 61.4 µM ^d^
**106** (2S)-5,7-dihydroxy-8,20-dimethoxyflavanone	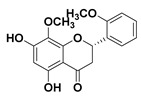		IC_50_ = 25.1 µM ^c^
**107** (2S)-5,20,50-trihydroxy-7, 8-dimethoxyflavanone	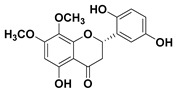		IC_50_ = 11.6 µM ^c^
**108** Caffeic acid phenylamide (CAPA)	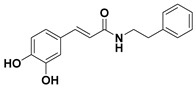	IC_50_ = 6.9 µM ^a^ IC_50_ = 60.3 µM ^e^	

^a^ bovine liver ARG; ^b^ rat liver ARG; ^c^ rat kidney ARG; ^d^ murine kidney ARG; ^e^ human ARG.
